# Oligomeric cystatin C supports the immunosuppressive activity of myeloid cells through interaction with inhibitory receptors

**DOI:** 10.1038/s41392-025-02462-x

**Published:** 2025-11-14

**Authors:** Chengcheng Zhang, Yubo He, Xiaoye Liu, Jingjing Xie, Meng Fang, Xing Yang, Ryan Huang, Qi Lou, Bufan Li, Ankit Gupta, Cheryl Lewis, Marc I. Diamond, Ningyan Zhang, Zhiqiang An, Cheng Cheng Zhang

**Affiliations:** 1 Department of Physiology, University of Texas Southwestern Medical Center, Dallas, TX USA; 2 Center for Alzheimer’s and Neurodegenerative Diseases, University of Texas Southwestern Medical Center, Dallas, TX USA; 3 Harold C. Simmons Comprehensive Cancer Center, University of Texas Southwestern Medical Center, Dallas, TX USA; 4 Peter O’Donnell Jr. Brain Institute, University of Texas Southwestern Medical Center, Dallas, TX USA; 5 Texas Therapeutics Institute, Brown Foundation Institute of Molecular Medicine, University of Texas Health Science Center, Houston, TX USA

**Keywords:** Tumour immunology, Tumour immunology

## Abstract

Amyloid proteins are linked to various diseases; however, their functional roles in immunity and cancer remain unclear. Here, we establish a direct link between oligomeric cystatin C—a cysteine cathepsin inhibitor and a well-characterized amyloidogenic protein—within the tumor microenvironment and the immune inhibitory receptors LILRB2 and LILRB5 on myeloid cells. We demonstrated that human LILRB2 and LILRB5, along with their murine counterpart PIRB, serve as functional receptors for cystatin C oligomers. Engagement of these inhibitory receptors by oligomeric cystatin C enhances the immunosuppressive activity of myeloid cells, leading to T-cell suppression and tumor progression. Deletion of the *CST3* gene, which encodes cystatin C, in host mice and tumor cells impaired tumor growth, whereas its overexpression accelerated cancer progression in LILRB2 and LILRB5 transgenic mice. Mechanistically, cystatin C–LILRB2 signaling is driven by both canonical phosphatases and the enhanced TGF-β pathway. Additionally, we identified interactions between LILRB receptors and transthyretin oligomers, another amyloid linked to transthyretin amyloidosis, suggesting a broader paradigm of amyloid–LILRB interactions. Our findings reveal an unexpected role of oligomeric cystatin C in enhancing myeloid cell immunosuppression, expand the functional spectrum of amyloid proteins and underscore the importance of these proteins in immune evasion and cancer development.

## Introduction

Traditionally, amyloid proteins have been studied primarily in the context of protein misfolding and aggregation disorders such as Alzheimer’s disease, Parkinson’s disease, and prion diseases. However, a growing body of evidence suggests that amyloid proteins are not solely pathological byproducts of misfolded protein deposition but may act as modulators of cellular activities and tissue homeostasis^1^. The roles of amyloid proteins in immunity and cancer development are complex and multifaceted. Researches revealed that amyloid proteins can regulate immune responses by interacting with different immune receptors. For example, amyloid β (Aβ), which is associated with Alzheimer’s disease pathology, has been shown to activate innate immune sensors Toll-like receptors (TLRs) and NOD-like receptors (NLRs), thereby leading to inflammatory cascades and microglial activation^2^. Conversely, Aβ has also been reported to interact with immune inhibitory receptors, leading to suppression of immune cell activity and highlighting its context-dependent immunomodulatory capacity^3^. Beyond neurodegeneration, amyloid proteins also appear to play roles in cancer biology. Amyloid precursor protein (APP), for example, is upregulated in advanced breast cancer and has been associated with increased cancer cell proliferation, migration, and invasion^4^. Similarly, elevated serum amyloid A (SAA) levels are strongly correlated with poorer overall survival in certain cancers, especially renal cell carcinoma and digestive cancer^5^. In addition to soluble amyloid peptides, extracellular amyloid fibrils have emerged as important regulators of the tumor microenvironment (TME). These fibrils have been shown to modulate mechanosignaling and influence the physical and biochemical properties of tumor niches in both melanoma and pancreatic ductal adenocarcinoma cells, thereby promoting tumor cells migration and invasion^6^. Interestingly, not all amyloid proteins promote tumor growth. Some amyloid proteins, including amyloid-β (Aβ), islet amyloid polypeptide (IAPP), and calcitonin (CT), can suppress pancreatic cancer cell proliferation in a sequence-, concentration-, and aggregation-dependent manner^7^. These contrasting findings underscore the multifaceted nature of amyloid proteins in cancer and immunity and highlight the critical need for further research to fully elucidate the relationships among amyloid proteins, immunity, and cancer.

Cystatin C, encoded by the *CST3* gene, is a potent cysteine cathepsin inhibitor and a well-characterized amyloidogenic protein associated with hereditary cystatin C amyloid angiopathy (HCCAA), an autosomal dominant disorder caused by amyloid deposition in cerebral vasculature^8^. Epidemiological studies and large-scale data analyses have linked elevated cystatin C expression to increased all-cause and cancer-specific mortality across diverse populations^9,10^. While cystatin C has been reported to promote tumor growth and therapy resistance by enhancing cancer cell survival during radiotherapy and facilitating tumor repopulation^11^, it has also demonstrated tumor-suppressive properties, mainly through its inhibitory action on cysteine cathepsins, enzymes that regulate extracellular matrix (ECM) degradation and remodeling, thereby inhibiting tumor cells invasion and metastasis^12^. Given these divergent roles, understanding the precise function of cystatin C in cancer biology has become an important area of investigation.

TME is a highly dynamic and heterogeneous system composed of immune cells, stromal cells, endothelial cells, soluble factors, and ECM components. Within this ecosystem, immunosuppressive myeloid cells have emerged as key regulators of immune evasion. Their accumulation and activation are strongly correlated with reduced efficacy of immunotherapies and poor prognosis across various cancers, such as colorectal cancer, pancreatic cancer, glioblastoma, breast cancer, liver cancer, head and neck cancer, lung cancer, and prostate cancer^13^. Among the key regulators of myeloid cell immunosuppressive function are the leukocyte immunoglobulin-like receptor subfamily B (LILRB) proteins. The LILRB proteins are type I transmembrane receptors predominantly expressed on hematopoietic cells^14–20^. They contain immunoreceptor tyrosine-based inhibitory motifs (ITIMs) that can recruit the tyrosine phosphatases SHP-1 and SHP-2 or the inositol phosphatase SHIP, and function as immune checkpoint molecules, playing immunosuppressive roles in human immunity and cancer development^20^. LILRBs are expressed predominantly on myeloid cells and their murine relatives—PirB and gp49B1^21,22^—differ in both expression patterns and ligand specificity. Consequently, knockout mouse models may not be optimal for elucidating the biological significance of LILRBs. Researches, including works from our laboratory, have increasingly highlighted the roles of LILRB family members in both hematologic malignancies and solid cancers^23–37^. Despite these advances, the precise immunosuppressive mechanisms mediated by LILRBs in cancer remain insufficiently understood.

Here, we demonstrate that cystatin C undergoes oligomerization within the TME. These oligomeric cystatin C directly bind to the inhibitory receptors LILRB2 and LILRB5 on myeloid cells, thereby significantly enhancing their immunosuppressive activity both in vitro and in vivo. This interaction leads to suppression of T-cell proliferation and activation, ultimately accelerating tumor growth and progression. Mechanistically, the immunosuppressive effects mediated by the cystatin C–LILRB2 axis are not only ITIM- and SHP-dependent but also driven, at least in part, by increased TGF-β signaling in myeloid cells. These findings open new promising therapeutic avenues, supporting the development of strategies that target cystatin C oligomerization and block LILRB2 and LILRB5 signaling for treating cystatin C-associated cancers. Notably, we also observed strong interactions between LILRB2 and LILRB5 receptors and transthyretin (TTR) oligomers, another well-characterized amyloid protein implicated in transthyretin amyloidosis, suggesting a broader role for amyloid–LILRB interactions in amyloid-related diseases.

## Results

### Oligomeric cystatin C binds to the inhibitory receptors LILRB2 and LILRB5

To investigate the potential role of cystatin C in cancer development, we measured circulating serum concentrations of cystatin C in healthy donors and cancer patients (Supplementary Table 1). Among the cancer patients surveyed, the majority presented elevated serum cystatin C levels compared with those of healthy controls (Supplementary Fig. 1a). Additionally, total soluble amyloid oligomers were detected in the serum samples of eight cancer patients (Supplementary Fig. 1b). We further analyzed the oligomerization status of cystatin C in sixteen melanoma tumor tissues under nonreducing conditions (Supplementary Table 2). Distinct patterns were observed: in 56.25% of the samples (9 out of 16), both monomeric and oligomeric forms of cystatin C were detected; in 31.25% of the samples (5 out of 16), the monomeric form predominated; and in the remaining 12.50% (2 out of 16), oligomeric forms were the major species (Supplementary Fig. 1c), suggesting that oligomeric cystatin C exists in tumor tissues, albeit with variable abundance across samples.

**Fig. 1:**
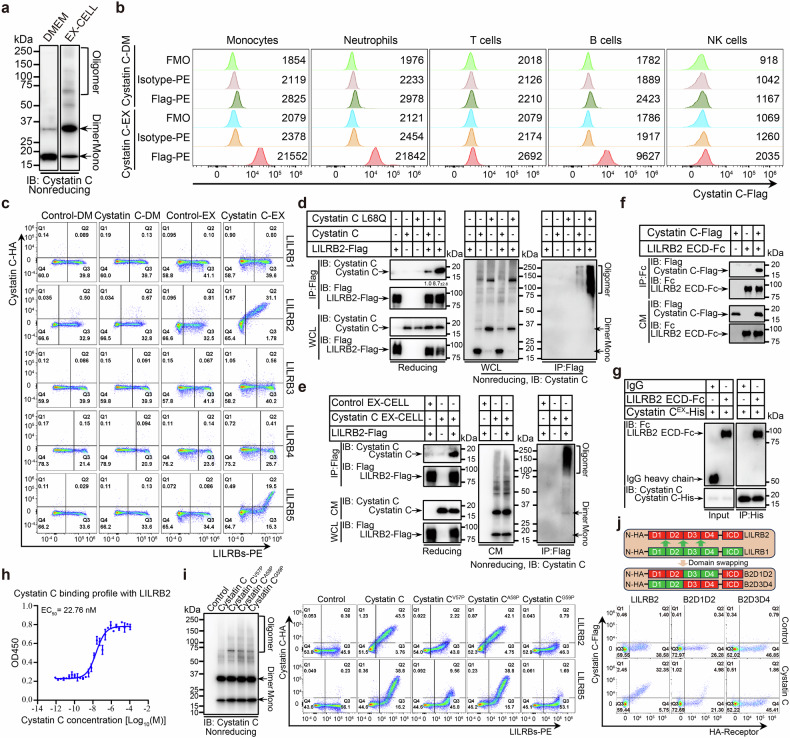
Identification of LILRB2 and LILRB5 as receptors for oligomeric cystatin C. **a** Western blot analysis of cystatin C in DMEM- or EX-CELL-conditioned medium from HEK293T cells expressing cystatin C via an anti-cystatin C monoclonal antibody under nonreducing conditions. **b** Representative flow cytometry histograms showing the binding of cystatin C-Flag in DMEM- or EX-CELL-conditioned medium to various primary immune cell populations from the peripheral blood of healthy donors. FMO (fluorescence minus one) and isotype antibodies served as negative controls. The mean fluorescence intensities (MFIs) are indicated. **c** Flow cytometry analysis of cystatin C-HA in DMEM or EX-CELL-conditioned medium binding to HEK293T cells expressing LILRBs. **d** Co-IP assay results showing that the cystatin C oligomers bind to LILRB2 in cotransfected HEK293T cells. Band intensities were quantified relative to those of the input and are presented as the means ± SD. *n* = 3 biological replicates. **e** Co-IP assays revealed that cystatin C oligomers bind to LILRB2 on the surface of HEK293T cells expressing LILRB2 following incubation with cystatin C-containing EX-CELL supernatant. **f** Co-IP assays revealed that cystatin C binds to the ECD of LILRB2 in EX-CELL-conditioned medium (CM) from cotransfected HEK293T cells. **g** Pull-down assays revealed that cystatin C^EX^ directly binds to the LILRB2 ECD. **h** Titration curves of cystatin C^EX^ binding to LILRB2 ECD-Fc measured by ELISA. The data are presented as the means ± SD. **i** Left: Western blot analysis of WT and mutated cystatin C in EX-CELL-conditioned medium from HEK293T cells expressing cystatin C via an anti-cystatin C monoclonal antibody under nonreducing conditions. Right: Flow cytometry analysis of WT and mutated cystatin C-HA in EX-CELL-conditioned medium bound to HEK293T cells expressing LILRB2 or LILRB5. **j** Top: Schematic diagram showing the design of the LILRB2 domain-swapping mutants. The B2D1D2 and B2D3D4 mutants were generated by replacing the D3D4 and D1D2 domains of LILRB2 with the corresponding domains from LILRB1, respectively. Bottom: Flow cytometry analysis of cystatin C-Flag in EX-CELL-conditioned medium bound to HEK293T cells expressing chimeric LILRB2. See also Supplementary Figs. 2, 3

To examine the function of potential oligomeric cystatin C in the TME, we analyzed the aggregation states of commercial recombinant cystatin C (SinoBiological, hereafter referred to as cystatin C^Sino^) under nonreducing conditions and found that it exists only as a monomer (Supplementary Fig. 2a). Next, we collected conditioned media from *CST3*-transfected HEK293T cells grown under different culture conditions. In Dulbecco’s modified Eagle’s medium (DMEM), cystatin C is predominantly monomeric, with a small fraction existing as dimers but no detectable oligomers. However, in EX-CELL-conditioned medium, monomeric cystatin C levels were reduced, whereas dimer and oligomer levels were increased, suggesting that cystatin C undergoes dimerization and oligomerization in this environment (Fig. 1a).

To determine whether cystatin C interacts with immune cells, we performed flow cytometry analysis. While cystatin C from DMEM-conditioned medium did not bind immune cells, EX-CELL-derived cystatin C exhibited significant binding to monocytes and neutrophils, with moderate binding to B cells (Fig. 1b). These findings suggest that cystatin C oligomers may engage immune cell receptors, thereby modulating downstream signaling. A genome-wide screen^33,34^ identified leukocyte Ig-like receptor subfamily B (LILRB) members as potential interacting partners for cystatin C. The LILRB subfamily consists of five members: LILRB1 to LILRB5. To validate the interaction between cystatin C and LILRBs, we assessed the binding of cystatin C to HEK293T cells expressing LILRBs via flow cytometry. Compared with monomeric cystatin C in DMEM, EX-CELL-derived cystatin C exhibited stronger binding to LILRB2- and LILRB5-expressing cells (Fig. 1c). Notably, mouse cystatin C displayed similar binding patterns, indicating a conserved interaction mechanism across species (Supplementary Fig. 2b, c).

We further confirmed the interaction between cystatin C and LILRB2/LILRB5 via coimmunoprecipitation (co-IP) assays. Cotransfection of HEK293T cells with *cystatin C* or the amyloidogenic *L68Q* mutant (associated with hereditary cystatin C amyloid angiopathy (HCCAA)) revealed that L68Q cystatin C interacts more strongly with LILRB2 and LILRB5 than wild-type cystatin C does, with oligomers being the predominant binding form (Fig. 1d and Supplementary Fig. 2d). The incubation of HEK293T cells expressing LILRB2 or LILRB5 with EX-CELL-derived cystatin C further confirmed this interaction (Fig. 1e and Supplementary Fig. 2e). Additionally, the cystatin C oligomers bound to the extracellular domains (ECDs) of LILRB2 and LILRB5 fused to IgG-Fc, reinforcing their direct interaction (Fig. 1f and Supplementary Fig. 2f).

To confirm direct binding, we purified cystatin C from EX-CELL medium (cystatin C^EX^; Supplementary Fig. 2g) and observed that cystatin C^EX^, but not cystatin C^Sino^, bound to LILRB2- and LILRB5-expressing cells (Supplementary Fig. 2h). In vitro pull-down assays using purified cystatin C^EX^ and LILRB2-Fc or LILRB5-Fc further validated this interaction (Fig. 1g and Supplementary Fig. 2i). ELISAs were used to determine the binding affinities, with EC50 values of ~22.76 nM and 1.18 μM for LILRB2 and LILRB5, respectively (Fig. 1h and Supplementary Fig. 2j). Biolayer interferometry also verified the direction interactions between cystatin C^EX^, but not cystatin C^Sino^, and LILRB2 and LILRB5 (Supplementary Fig. 2k).

Previous studies have shown that as cystatin C oligomerizes, structural changes occur primarily within the L1 loop, which acts as a molecular hinge during domain swapping^38^. To identify the structural motifs responsible for LILRB2 and LILRB5 binding, we introduced proline substitutions into the L1 loop turn region (*Val57-Ala58-Gly59*). While these mutations modestly accelerated oligomerization, the V57P and G59P substitutions significantly reduced LILRB2 and LILRB5 binding, whereas A58P had a minimal impact (Fig. 1i). These findings suggest that the L1 loop plays a critical role in the recognition of the LILRB2 and LILRB5 receptors.

LILRB2 and LILRB5 contain four extracellular immunoglobulin (Ig) domains. To determine which domains mediate cystatin C binding, we employed a chimeric LILRB2 construct in which the D3D4 (Ig3--Ig4) domains were replaced with those from LILRB1 (B2D1D2) and vice versa (B2D3D4; Fig. 1j). Consistent with previous reports that LILRB2 binds β-amyloid oligomers via its D1D2 domain^3^, our flow cytometry analysis confirmed that the N-terminal D1D2 domain of LILRB2 is critical for cystatin C binding, whereas the D3D4 domain has minimal affinity (Fig. 1j). In contrast, for LILRB5, the C-terminal D3D4 domain—rather than the NK-terminal D1D2 domain—was essential for cystatin C binding (Supplementary Fig. 2l). These results suggest that LILRB2 and LILRB5 share a common ligand, but their ligand-binding mechanisms differ.

Paired immunoglobulin-like receptor B (PIRB) and gp49B1 are the murine relatives of LILRB^21,22^. To assess whether these receptors also recognize cystatin C, we tested their interactions with human and mouse cystatin C oligomers. Both human and mouse EX-CELL-derived cystatin C bound robustly to PIRB-expressing HEK293T cells but not to gp49B1-expressing cells (Supplementary Fig. 3a, b). The incubation of *PIRB*-transfected HEK293T cells with mouse cystatin C from EX-CELL medium further validated the PIRB-cystatin C interaction (Supplementary Fig. 3c). Moreover, compared with wild-type mice, *PIRB−/−* mice presented a significant reduction in cystatin C oligomer binding to monocytes and neutrophils, suggesting that PIRB is a receptor for cystatin C oligomers on myeloid cells (Supplementary Fig. 3d). Collectively, these results demonstrate that oligomeric cystatin C selectively binds to human LILRB2 and LILRB5, as well as mouse PIRB, establishing a conserved mechanism of immunosuppressive receptor engagement.

### Oligomeric cystatin C induces downstream signaling via LILRB2 and LILRB5 inhibitory receptors

**Fig. 2:**
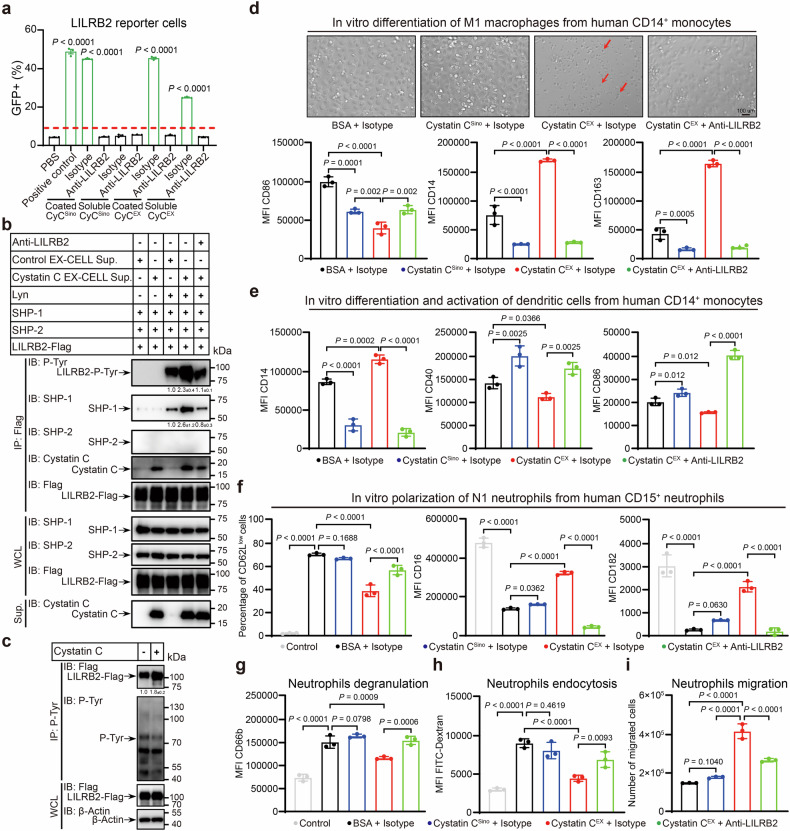
Cystatin C oligomers activate the LILRB2 inhibitory receptor to inhibit the differentiation or polarization of macrophages, dendritic cells, and neutrophils. **a** Percentages of LILRB2 reporter cells activated on plates coated with or exposed to soluble commercial cystatin C^Sino^ (20 μg/mL) or cystatin C^EX^ (20 μg/mL) in the presence or absence of an anti-LILRB2 (20 μg/mL) blocking antibody. *n* = 3 biological replicates. The threshold of activation is defined as twice that of the negative control treatment. **b** Co-IP analysis of LILRB2-specific phosphotyrosine (P-Tyr) and SHP-1/2 recruitment in HEK293T cells cotransfected with LILRB2-Flag, Lyn, SHP-1, and SHP-2 plasmids, with or without anti-LILRB2 antibody treatment, followed by incubation with cystatin C EX-CELL-conditioned medium. Band intensities were quantified relative to the input and are presented as the means ± SD. *n* = 3 biological replicates. **c** Co-IP analysis of LILRB2-specific P-Tyr in stable THP-1-LILRB2 cells after a 10-min incubation on plates coated with cystatin C (20 μg/mL). β-actin served as the internal control. Band intensities were quantified relative to the input and are presented as the means ± SD. *n* = 3 biological replicates. **d** M1 macrophages were differentiated in vitro from CD14^+^ monocytes isolated from fresh human PBMCs. The cells were treated with cystatin C^EX^ and anti-LILRB2 antibodies from day 0, when differentiation started. Top: Cell morphology on day 6, Scale bar = 100 µm. Bottom: MFIs of CD86, CD14, and CD163 were measured via flow cytometry on day 6. *n* = 3 biological replicates. **e** Dendritic cells were differentiated in vitro from CD14^+^ monocytes isolated from fresh human PBMCs. Cystatin C^EX^ and anti-LILRB2 antibodies were included in the differentiation from day 0. The MFIs of CD14, CD40, and CD86 were measured on day 8 via flow cytometry, which was 2 days after stimulation. *n* = 3 biological replicates. **f**, **g** Primary human neutrophils were incubated for 24 h in the presence of an N1 polarization cocktail, including the pan-caspase inhibitor Q-VD-Oph. The cells were treated with cystatin C^EX^ and anti-LILRB2 antibodies beginning on day 0, when polarization started. The percentage of CD62L^low^ cells and the MFIs of CD16, CD182 and CD66b were measured via flow cytometry. Neutrophils that were treated with only Q-VD-Oph served as controls. *n* = 3 biological replicates. **h** Primary human neutrophils were cultured in serum-free RPMI 1640 medium under the indicated treatment conditions for 12 h in the presence of the pan-caspase inhibitor Q-VD-Oph. The cells were then incubated with 0.1 mg/mL FITC-conjugated dextran at 37 °C for 45 min to allow endocytosis. The MFI of FITC was measured via flow cytometry. A parallel group incubated at 4 °C served as a control for nonspecific binding and passive uptake. *n* = 3 biological replicates. **i** Primary human neutrophils were pretreated with either an isotype control or anti-LILRB2 blocking antibody in serum-free RPMI 1640 medium for 4 h at 37 °C in the presence of the pan-caspase inhibitor Q-VD-Oph. Neutrophil suspensions were added to the upper chambers of transwell inserts, and the indicated serum-free RPMI 1640 medium was added to the lower chambers. The cells were incubated at 37 °C for 17 h. Neutrophils that migrated to the lower chambers were collected and quantified by flow cytometry. *n* = 3 biological replicates. The data are presented as the means ± SD. *P* values were determined by one-way ANOVA with Dunnett’s multiple comparisons test (**a**) or Holm‒Sidak’s multiple comparisons test (**d**–**i**). See also Supplementary Fig. 4

To determine whether cystatin C oligomers function as ligands for LILRB2- and LILRB5-mediated signaling, we utilized a sensitive chimeric receptor reporter system^23^. In this system, the extracellular domains (ECDs) of LILRBs are fused to the transmembrane and intracellular domains of PILRβ, allowing ligand-induced activation of the LILRB ECD to recruit ZAP70 or Syk kinase to the immunoreceptor tyrosine-based activation motif (ITAM) of the adaptor protein DAP12, ultimately triggering GFP expression under an NFAT-responsive promoter (Supplementary Fig. 4a). Both LILRB2 and LILRB5 reporter cells were activated by immobilized commercial cystatin C^Sino^ or cystatin C^EX^ (which promotes protein clustering on the plastic surface through tethering). However, only soluble cystatin C^EX^, but not cystatin C^Sino^, effectively induced LILRB2 and LILRB5 reporter cell activation, further suggesting that oligomeric cystatin C binds and activates LILRB2 and LILRB5 (Fig. 2a and Supplementary Fig. 4b). This activation was abrogated by anti-LILRB2 and anti-LILRB5 blocking antibodies, confirming the specificity of the interaction between cystatin C and its receptors (Fig. 2a and Supplementary Fig. 4b).

Next, we investigated whether cystatin C oligomers activate LILRB2 and LILRB5 in HEK293T cells reconstituted with LILRB signaling components^25,32^. Upon ligand binding, the tyrosine residues within the immunoreceptor tyrosine-based inhibitory motifs (ITIMs) of LILRBs become phosphorylated, facilitating the recruitment of the tyrosine phosphatases SHP-1 and SHP-2 or the inositol phosphatase SHIP, ultimately leading to immune suppression. Cotransfection of HEK293T cells with *LILRB2-Flag*, *Lyn kinase*, and *SHP-1/SHP-2*, followed by incubation with EX-CELL-conditioned cystatin C medium, revealed that cystatin C oligomers induce LILRB2-specific tyrosine phosphorylation and promote SHP-1 recruitment. This activation was effectively reversed by anti-LILRB2 blocking antibodies (Fig. 2b). Similar results were observed in the HEK293T-LILRB5 system (Supplementary Fig. 4c).

To further confirm the ability of cystatin C oligomers to activate LILRB2 and LILRB5, we generated THP-1-derived monocyte cell lines stably expressing LILRB2 or LILRB5. cystatin C-coated plates consistently induced tyrosine phosphorylation of LILRB2 and LILRB5 in these cells (Fig. 2c and Supplementary Fig. 4d). Together, these results establish oligomeric cystatin C as a functional extracellular ligand that binds and activates the inhibitory receptors LILRB2 and LILRB5, promoting downstream immunosuppressive signaling.

### Oligomeric cystatin C enhances LILRB2-dependent immunosuppressive myeloid cell activity and T-cell suppression

LILRB2 is expressed predominantly on myeloid cells. To ensure the suitability of cystatin C^EX^ for use in primary cell assays, we first assessed and confirmed its minimal endotoxin content (Supplementary Fig. 4e). To investigate whether oligomeric cystatin C influences macrophage differentiation and polarization via LILRB2, freshly isolated CD14⁺ monocytes were differentiated into M1 macrophages via GM-CSF, with or without cystatin C^EX^ in the presence or absence of anti-LILRB2 blocking antibodies. Compared with BSA-treated controls, cystatin C^EX^ significantly inhibited the GM-CSF-induced round and flattened morphology characteristic of M1 macrophages after six days of differentiation, an effect reversed by LILRB2 blockade (Fig. 2d). Surface marker expression analysis further supported these observations, as cystatin C^EX^ suppressed CD86 expression, maintained CD14 expression, and prevented the downregulation of CD163, which are hallmarks of M1 macrophage differentiation. These effects were also reversed by anti-LILRB2 treatment (Fig. 2d). Additionally, the mRNA expression levels of the immunosuppressive genes *IL-10* and *NOS2* were also consistent with these findings (Supplementary Fig. 4f). These results indicate that cystatin C oligomers inhibit M1 macrophage differentiation via LILRB2.

We next assessed whether oligomeric cystatin C affects monocyte differentiation into anti-inflammatory M2a macrophages. Compared with BSA-treated controls, cystatin C^EX^ did not alter the elongated, loosely adherent morphology of M2a macrophages induced by M-CSF and IL-4 (Supplementary Fig. 4g). Additionally, upon further polarization with LPS, cystatin C^EX^ did not change CD163 or CD206 surface expression levels, but it significantly inhibited the expression of the M1 markers CD80 and CD86, indicating reduced M1 functional output. This suppression was reversed by LILRB2 blockade, further supporting the role of LILRB2 in mediating the effects of cystatin C (Supplementary Fig. 4g).

Since the cross-presentation of antigens by dendritic cells (DCs) plays a critical role in priming antitumor CD8⁺ T cells, we examined whether cystatin C oligomers affect monocyte-derived DC differentiation and activation. Differentiation into DCs is typically characterized by loss of CD14 expression and upregulation of DC markers such as CD40 and CD86. However, cystatin C^EX^ treatment inhibited the downregulation of CD14 and suppressed the upregulation of CD40 and CD86 (Fig. 2e). These inhibitory effects were reversed by anti-LILRB2, confirming that cystatin C oligomers impair DC differentiation through LILRB2 (Fig. 2e).

Neutrophils, which are traditionally known for their antimicrobial function, exhibit plasticity in cancer, adopting either an antitumorigenic (N1) or a protumorigenic (N2) phenotype. To determine whether cystatin C oligomers regulate neutrophil polarization, freshly isolated CD15⁺ neutrophils were treated with an N1-inducing cocktail (LPS, IFN-γ, and IFN-β) in the presence or absence of anti-LILRB2 blocking antibodies. To prolong the lifespan of neutrophils, cells were treated with the pan-caspase inhibitor Q-VD-Oph. Flow cytometry analysis revealed that N1 polarization, which is characterized by CD62L^neg/low^ expression and CD16 downregulation, was significantly inhibited by cystatin C^EX^, and this effect was reversed by LILRB2 blockade (Fig. 2f). Additionally, cystatin C^EX^ treatment significantly upregulated the expression of CXCR2 (CD182), a marker of N2 neutrophils, further supporting its role in promoting an immunosuppressive neutrophil phenotype (Fig. 2f). Consistent with these findings, functional assays demonstrated that cystatin C^EX^ significantly inhibited neutrophil degranulation and endocytosis while promoting neutrophil migration (Fig. 2g–i).

**Fig. 3:**
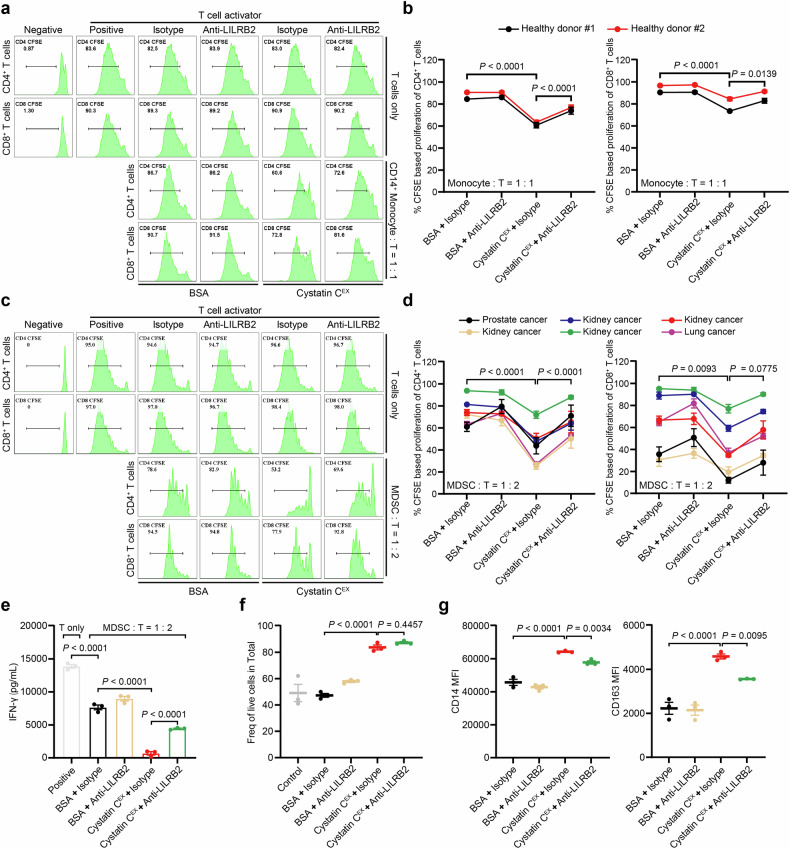
Cystatin C oligomers enhance the immunosuppressive activity of monocytes and MDSCs in healthy individuals and cancer patients, respectively. **a** Representative flow cytometry histograms showing that cystatin C oligomers enhance the T-cell suppressive activity of monocytes in a CSFE assay. CD14^+^ monocytes from healthy donors were cocultured with CFSE-stained autologous T cells for 5 days. Cystatin C^EX^ and anti-LILRB2 antibodies were included in the coculture system from day 0. **b** Percentages of proliferative CD4^+^ and CD8^+^ T cells in the monocyte:T coculture system under the indicated treatment conditions. **c** Representative flow cytometry histograms showing that cystatin C oligomers enhance the T-cell suppressive activity of MDSCs in a CSFE assay. MDSCs from cancer patients were cocultured with CFSE-stained autologous T cells for 5 days. Cystatin C^EX^ and anti-LILRB2 antibodies were included in the coculture system from day 0. **d** Percentage of proliferative CD4^+^ and CD8^+^ T cells in the MDSC:T coculture system under the indicated treatment conditions. **e** ELISA analysis of IFN-γ secretion from the coculture system under the indicated treatment conditions. *n* = 3 biological replicates. **f**, **g** Enriched MDSCs from cancer patients were cultured for 2 days. Cystatin C^EX^ and anti-LILRB2 antibodies were included from day 0. The percentage of live cells (**f**) and the MFIs of surface CD14 and CD163 (**g**) were measured via flow cytometry. *n* = 3 biological replicates. The data are presented as the means ± SD. *P* values were determined via one-way ANOVA with Holm‒Sidak’s multiple comparisons test. See also Supplementary Fig. 5

To determine whether cystatin C oligomers influence T-cell proliferation by modulating myeloid cell activity, freshly isolated CD14⁺ monocytes and autologous T cells from healthy donors were cocultured with cystatin C^EX^ in the presence or absence of anti-LILRB2 blocking antibodies. While neither monocytes nor cystatin C^EX^ alone affected T-cell proliferation (Fig. 3a and Supplementary Fig. 5a), their combined presence significantly suppressed T-cell proliferation, an effect that was partially reversed by LILRB2 blockade (Fig. 3a, b). These results suggest that cystatin C oligomers reprogram monocytes into an immunosuppressive state via LILRB2, ultimately inhibiting T-cell proliferation.

To further examine the role of cystatin C oligomers in cancer-associated immunosuppression, we isolated myeloid-derived suppressor cells (MDSCs) and autologous T cells from peripheral blood samples of patients with prostate, kidney, and lung cancer. As expected, patient-derived MDSCs suppressed the proliferation and function of autologous T cells in a coculture system (Fig. 3c–e and Supplementary Fig. 5b), which is consistent with their known immunosuppressive activity^31,32^. Notably, cystatin C^EX^ further enhanced the MDSC-mediated suppression of T-cell proliferation and function (Fig. 3c–e and Supplementary Fig. 5b). This immunosuppressive effect was partially reversed by anti-LILRB2 treatment, reinforcing the role of cystatin C oligomers in promoting MDSC function (Fig. 3c–e). To further clarify the effect of cystatin C oligomers on MDSC function, we treated patient-derived MDSCs with cystatin C^EX^ in the presence or absence of anti-LILRB2 blocking antibodies. Cystatin C^EX^ significantly increased MDSC viability in vitro (Fig. 3f). In addition, the surface expression of the immunosuppressive markers CD14 and CD163 was increased following cystatin C^EX^ treatment, which was partially reversed by anti-LILRB2 blockade (Fig. 3g). Together, these results indicate that cystatin C oligomers support the immunosuppressive activities of primary myeloid cells in vitro through the LILRB interaction.

### *CST3* silencing inhibits tumor growth in mouse models

To investigate whether cystatin C supports the immunosuppressive activity of myeloid cells in vivo, we first evaluated the oligomerization of mouse cystatin C in the TME. In the B16-F10 melanoma model, compared with adjacent normal skin tissue, cystatin C underwent moderate oligomerization during tumor progression (Supplementary Fig. 6a, b). Next, we generated a *CST3* knockout (*cystatin C*−*/−*) mouse strain on the C57BL/6 background. In wild-type (WT) mice, *CST3* mRNA was widely expressed, with particularly high levels in the brain (Supplementary Fig. 6c). Soluble cystatin C was also detected in mouse blood (Supplementary Fig. 6d). In contrast, western blot and dot blot assays confirmed the absence of cystatin C protein in the brain, lung, muscle, heart, spleen, kidney, liver, and plasma of *cystatin C*−*/−* mice (Supplementary Fig. 6d, e). Despite this depletion, *cystatin C*−*/−* mice presented a normal appearance, organ size, and litter frequency, suggesting that cystatin C is not essential for overall development and growth. Furthermore, cystatin C deficiency had no detectable effect on immune cell populations in the peripheral blood (Supplementary Fig. 6f).

**Fig. 4:**
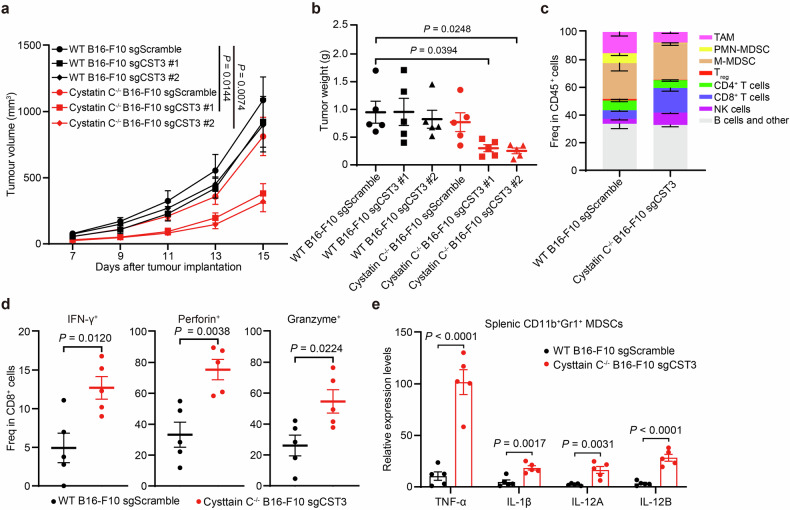
*Cystatin C*−*/−* mice exhibit significantly attenuated tumor growth. **a**, **b** *B16-F10 sgScramble* or *B16-F10 sgCST3* melanoma cells were inoculated subcutaneously into WT and *cystatin C*−*/−* mice. The tumor growth curves (A) and tumor weights (B) of each group (*n* = 5) are shown. **c** Percentages of the indicated immune cell types in *B16-F10 sgScramble* tumors from WT mice (*n* = 5) and *B16-F10 sgCST3* tumors *from cystatin C*−*/−* mice (*n* = 5) as measured by flow cytometry. **d** Percentage of IFN-γ^+^, perforin^+^, and granzyme^+^ CD8^+^ T cells from *B16-F10 sgScramble* tumors of WT mice (*n* = 5) and *B16-F10 sgCST3* tumors of *cystatin C*−*/−* mice (*n* = 5) as measured by flow cytometry. **e** RT‒qPCR analysis of TNF-α, IL-1β, IL-12A, and IL-12B cytokine expression in CD11b^+^Gr-1^+^ MDSCs sorted from the spleens of WT mice (*n* = 5) bearing *B16-F10 sgScramble* tumors or *cystatin C*−*/−* mice (*n* = 5) bearing *B16-F10 sgCST3* tumors. The data are presented as the means ± SEM. *P* values were determined by one-way ANOVA with Dunnett’s multiple comparisons test (**a** and **b**) or two-tailed Student’s *t* test (**d** and **e**). See also Supplementary Fig. 6

To assess the role of tumor-derived cystatin C, we used the CRISPR-Cas9 system to generate a *CST3*-knockout B16-F10 melanoma cell line (Supplementary Fig. 6g). MTS assays revealed that sgScramble and sgCST3 B16-F10 clones had equivalent proliferative activities in vitro (Supplementary Fig. 6h). We then subcutaneously inoculated these cells into both WT and *cystatin C*−*/−* mice. While tumor growth kinetics were similar between sgScramble and sgCST3 B16-F10 clones in WT mice, *cystatin C*−*/−* mice presented significantly reduced tumor growth and lower endpoint tumor weights for sgCST3, but not sgScramble, B16-F10 tumors (Fig. 4a, b).

To determine whether this altered tumor growth was associated with changes in the TME, we performed immune profiling of tumor tissues from *cystatin C*−*/−* mice. In the B16-F10 tumor model, cystatin C depletion led to a substantial reduction in tumor-associated macrophages (TAMs), polymorphonuclear myeloid-derived suppressor cells (PMN-MDSCs), and regulatory T cells (Tregs) but significantly increased the infiltration of CD8⁺ T cells and natural killer (NK) cells (Fig. 4c and Supplementary Fig. 6i). This shift in immune composition indicates that cystatin C depletion transforms the TME from protumor to antitumor.

Consistent with increased CD8⁺ T-cell infiltration, the percentages of IFN-γ⁺, perforin⁺, and granzyme⁺ CD8⁺ T cells were significantly greater in the TME of *cystatin C*−*/−* mice than in that of WT mice, suggesting enhanced T-cell cytotoxicity (Fig. 4d). Additionally, cytokine analysis of sorted splenic Mac1⁺Gr-1⁺ myeloid cells from tumor-bearing mice revealed that compared with WT controls, cystatin C deletion significantly increased the expression of proinflammatory cytokines, including TNF-α, IL-1β, and IL-12 (Fig. 4e). These findings demonstrate that cystatin C plays a key role in shaping an immunosuppressive TME by promoting the accumulation of immunosuppressive myeloid cells while inhibiting antitumor immune responses. *CST3* silencing disrupts these immunosuppressive effects, leading to enhanced T-cell cytotoxicity and suppressed tumor growth in vivo.

### *CST3* overexpression promotes tumor growth in LILRB2- and LILRB5-transgenic mice

**Fig. 5:**
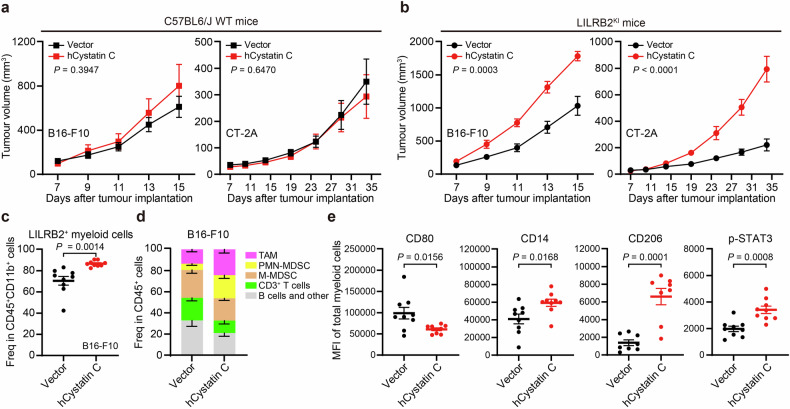
Forced expression of *CST3* promotes tumor growth in LILRB2^KI^ mice. **a** C57BL6/J WT mice were inoculated subcutaneously with *B16-F10-vector*, *B16-F10-hcystatin C*, *CT-2A-vector*, or *CT-2A-hcystatin C* cells. The tumor growth curves of each group (*n* = 8 or 9) are shown. **b** LILRB2^KI^ mice were inoculated subcutaneously with *B16-F10-vector*, *B16-F10-hcystatin C*, *CT-2A-vector*, or *CT-2A-hcystatin C* cells. The tumor growth curves of each group (*n* = 9 or 10) are shown. **c** Percentages of LILRB2^+^ myeloid cells in *B16-F10-vector* or *B16-F10-hcystatin C* tumors from LILRB2^KI^ mice, as measured via flow cytometry. **d** Percentage of indicated immune cell types in the *B16-F10-vector* or *B16-F10-hcystatin C* tumors of LILRB2^KI^ mice as measured by flow cytometry. **e** MFIs of the indicated surface or intracellular markers of myeloid cells from *B16-F10-vector* or *B16-F10-hcystatin C* tumors in LILRB2^KI^ mice were measured via flow cytometry. The data are presented as the means ± SEM. *P* values were determined via two-tailed Student’s *t* test. See also Supplementary Fig. 7

Given that LILRB family members are specific to primates, we employed myeloid-specific (*LysM-Cre*) LILRB2-transgenic (LILRB2^KI^) and LILRB5-transgenic (LILRB5^KI^) C57BL/6 mice to investigate the functional role of human cystatin C in activating LILRB2 and LILRB5 in vivo. We generated stable human cystatin C-expressing B16-F10 (*B16-F10-hcystatin C*) and CT-2A (*CT-2A-hcystatin C*) glioma cell lines (Supplementary Fig. 7a, b) and implanted them into LILRB2^KI^ or LILRB5^KI^ mice. As expected, forced expression of human cystatin C in B16-F10 or CT-2A cells significantly accelerated tumor growth in LILRB2^KI^ mice but not in WT mice (Fig. 5a, b). Additionally, substantial oligomerization of human cystatin C was observed in the TME of *B16-F10-hcystatin C* and *CT-2A-hcystatin C* tumors in LILRB2^KI^ mice (Supplementary Fig. 7c). Consistent with this tumor-promoting phenotype, flow cytometry analysis revealed that *B16-F10-hcystatin C* overexpression significantly increased the proportion of LILRB2⁺ myeloid cells among total myeloid cells in LILRB2^KI^ mice (Fig. 5c), indicating a more immunosuppressive TME. This finding was further supported by the increased presence of immunosuppressive tumor-associated macrophages (TAMs) and PMN-MDSCs, accompanied by a reduction in immunostimulatory T cells (Fig. 5d and Supplementary Fig. 7d).

Similarly, in the *CT-2A-hcystatin C* tumor model, immune profiling revealed increased accumulation of immunosuppressive myeloid cell populations, including TAMs, PMN-MDSCs, M-MDSCs, and Treg cells, compared with those in the *CT-2A-vec* control model. In contrast, immune cell subsets with antitumor activity, such as T cells and NK cells, were significantly reduced (Supplementary Fig. 7e).

Further analysis of myeloid cells in the *B16-F10-hcystatin C* model revealed significant downregulation of CD80 expression and upregulation of CD14 and CD206 expression, indicative of a tolerogenic myeloid phenotype (Fig. 5e). Additionally, intracellular staining revealed significantly elevated levels of phosphorylated STAT3, a key regulator of MDSC differentiation and immunosuppressive function (Fig. 5e).

Importantly, anti-LILRB2 treatment significantly delayed *B16-F10-hcystatin C* tumor progression in LILRB2^KI^ mice (Supplementary Fig. 7f). Furthermore, *B16-F10-hcystatin C* tumor growth was accelerated in LILRB5^KI^ mice (Supplementary Fig. 7g, h). These findings suggest that cystatin C enhances the immunosuppressive functions of tumor-associated myeloid cells through interaction with the LILRB2 and LILRB5 receptors in vivo.

### *CST3* overexpression promotes tumor growth in humanized mice

**Fig. 6:**
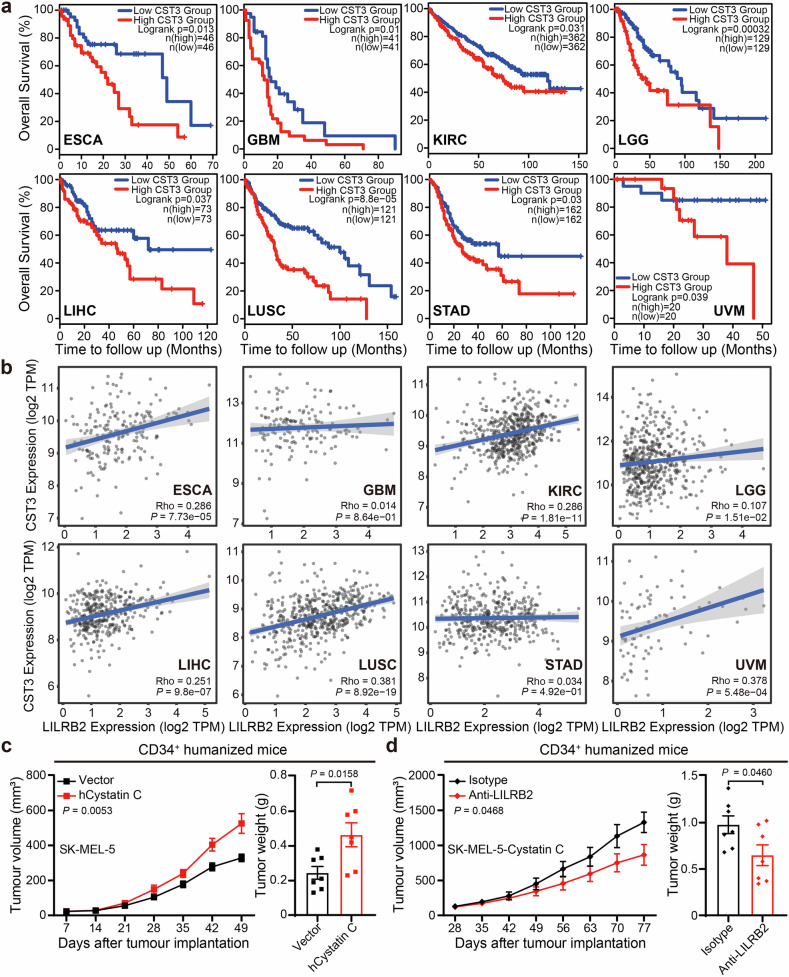
High expression of cystatin C in human cancers is associated with a poor prognosis. **a** Kaplan–Meier survival analysis of the correlations between *CST3* expression and overall survival in patients with esophageal cancer (ESCA), glioblastoma (GBM), kidney clear cell carcinoma (KIRC), lower grade glioma (LGG), liver cancer (LIHC), lung squamous cell carcinoma (LUSC), stomach cancer (STAD), and ocular melanoma (UVM). Patients were stratified by low levels of *CST3* (blue) or high levels of *CST3* (red) from the TCGA database. Statistical significance was calculated via the log-rank test. **b** Bioinformatic analysis of the correlation between *CST3* and LILRB2 expression across the indicated cancer types was performed via TIMER 2.0. For each tumor type, RNA-seq expression values (TPM, transcripts per million) from TCGA were log2-transformed, and Spearman’s rank correlation coefficients (Rho) were calculated. Statistical significance was assessed by *P* values provided by TIMER 2.0, with multiple-testing correction across cancer types via the Benjamini–Hochberg method. Correlations were defined as positive when Rho > 0 with *P* < 0.05 and negative when Rho < 0 with *P* < 0.05. **c** *SK-MEL-5-vector* or *SK-MEL-5-hcystatin C* melanoma cells were inoculated subcutaneously into humanized mice. Tumor growth curves and tumor weights in each group (*n* = 7) are shown. **d** Humanized mice were inoculated subcutaneously with *SK-MEL-5-hcystatin C* melanoma cells on day 0 and treated with isotype or anti-LILRB2 monoclonal antibody every 7 days from day 28 to day 70. Tumor growth curves and tumor weights in each group (*n* = 7) are shown. The data are presented as the means ± SEM. *P* values were determined via two-tailed Student’s *t* test (**c**, **d**). See also Supplementary Fig. 8

Human *CST3* mRNA and protein are ubiquitously expressed across normal tissues, with myeloid-derived cells identified as the primary contributors to extracellular cystatin C levels in healthy individuals, according to BioGPS tissue microarray and proteome analysis (Supplementary Fig. 8a). Single-cell RNA sequencing (scRNA-seq) data from 12 melanoma patients confirmed high *CST3* expression within the myeloid cell compartment, as well as ectopic expression in the tumor compartment^9^. In lung squamous cell carcinoma, elevated expression of the *CST3* gene in tumor-associated fibroblasts, an alternative source of cystatin C, is also associated with reduced responsiveness to immunotherapy and worse clinical outcomes^39^. Bioinformatic analysis of data from The Cancer Genome Atlas (TCGA) revealed a modest correlation between high *CST3* expression and poor overall survival across all cancer patients (*P* > 0.05, but with a trend toward significance; Supplementary Fig. 8b). Notably, *CST3* expression was negatively correlated with overall survival in patients with esophageal cancer, glioblastoma, kidney clear cell carcinoma, lower-grade glioma, liver cancer, lung squamous cell carcinoma, stomach cancer, and ocular melanoma (Fig. 6a). Moreover, *CST3* expression was significantly positively correlated with the expression of *LILRB2* or *LILRB5*, particularly *LILRB2*, in most of these cancer types, although these observed Spearman’s rho values were within the range typically considered weak correlations (Fig. 6b and Supplementary Fig. 8c). High *CST3* expression was also significantly associated with poor disease-free survival across all cancer patients (Supplementary Fig. 8b), further implicating cystatin C in adverse clinical outcomes.

To investigate the role of cystatin C in human tumor development, we established a humanized mouse model by transplanting human cord blood-derived CD34⁺ cells into NSG-SGM3 mice, as described previously^23,31^. After seven weeks, robust human CD45⁺ chimerism was detected in the peripheral blood of hCD34⁺ humanized mice (Supplementary Fig. 8d). Subsequently, human cystatin C was stably expressed in SK-MEL-5 (*SK-MEL-5-hcystatin C*) melanoma cells (Supplementary Fig. 8e), which were subsequently implanted subcutaneously into NSG-SGM3 and hCD34⁺ humanized mice. Forced expression of human cystatin C significantly accelerated tumor growth in hCD34⁺ humanized mice but not in NSG-SGM3 mice (Fig. 6c and Supplementary Fig. 8f), suggesting that the tumor-promoting effects of cystatin C depend on human immune cells.

To determine whether LILRB2 mediates the tumor-support effects of cystatin C, hCD34⁺ humanized mice bearing *SK-MEL-5-hcystatin C* tumors were treated with an anti-LILRB2 blocking antibody when the tumors reached 100–120 mm^3^. Compared with isotype control treatment, blockade of LILRB2 significantly delayed *SK-MEL-5-hcystatin C* tumor growth (Fig. 6d). These results suggest that targeting the cystatin C-LILRB2 interaction could represent a promising cancer immunotherapy strategy in humans.

### The cystatin C–LILRB2 axis enhances TGF-β signaling in myeloid cells

**Fig. 7:**
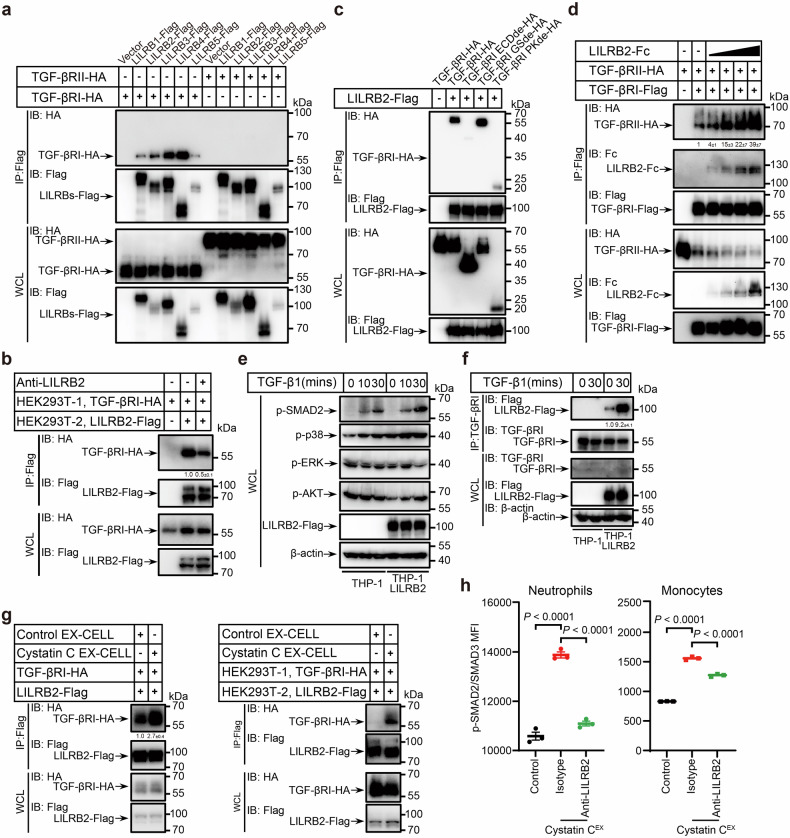
Cystatin C oligomers enhance TGF-β signaling in myeloid cells through the LILRB2 inhibitory receptor. **a** Co-IP assay showed that LILRBs preferentially bind to TGF-βRI rather than to TGF-βRII in cotransfected HEK293T cells, followed by normalization of the TGF-βRI and TGF-βRII protein levels. **b** Co-IP assay showing the *trans* interactions between LILRB2 and TGF-βRI in HEK293T cells expressing LILRB2-Flag or TGF-βRI-HA. Band intensities were quantified relative to the input and are presented as the means ± SD. *n* = 3 biological replicates. **c** Co-IP assays revealed that LILRB2 binds to the ECD of TGF-βRI in cotransfected HEK293T cells. **d** Co-IP assays revealed that LILRB2 promoted interactions between TGF-βRI and TGF-βRII in a dose-dependent manner in cotransfected HEK293T cells. Band intensities were quantified relative to the input and are presented as the means ± SD. *n* = 3 biological replicates. **e** Western blot analysis of p-SMAD2, p-p38, p-ERK, and p-AKT in THP-1 and THP-1-LILRB2 cells after overnight starvation and 30-min treatment with TGF-β1 (20 ng/mL). β-actin served as the internal control. **f** Co-IP assay showing the interactions between LILRB2 and TGF-βRI in THP-1-LILRB2 cells after overnight starvation and 30-min treatment with TGF-β1 (20 ng/mL). β-actin served as the internal control. Band intensities were quantified relative to the input and are presented as the means ± SD. *n* = 3 biological replicates. **g** Co-IP assays revealed that cystatin C in EX-CELL-conditioned medium enhances both the *cis* (left) and *trans* (right) interactions between LILRB2 and TGF-βRI in HEK293T cells. Band intensities were quantified relative to the input and are presented as the means ± SD. *n* = 3 biological replicates. **h** Primary neutrophils or monocytes were treated with cystatin C^EX^ and anti-LILRB2 antibodies overnight. The MFIs of intracellular p-SMAD2/SMAD3 were measured via flow cytometry after a 30-min treatment with TGF-β1 (20 ng/mL). *n* = 3 biological replicates. The data are presented as the means ± SD. *P* values were determined via one-way ANOVA with Holm‒Sidak’s multiple comparisons test. See also Supplementary Fig. 9

Myeloid-specific TGF-β signaling plays a critical role in immune evasion^40^, distinct from its tumor-suppressive effects observed in epithelial cells, fibroblasts, T cells, and tumor cells^41–44^. Given the well-established immunosuppressive role of LILRBs in myeloid cells, we investigated whether LILRBs interact with and modulate the TGF-β signaling pathway. TGF-β signals through a heteromeric complex composed of type I and type II serine/threonine kinase receptors, designated TGF-βRI and TGF-βRII, respectively^45^. Upon TGF-β binding, TGF-βRII phosphorylates and activates TGF-βRI, initiating downstream signaling cascades. Canonically, TGF-βRI phosphorylates SMAD2 and SMAD3, which then form a complex with SMAD4 and translocate to the nucleus to regulate gene expression^46^. In addition, TGF-β can engage noncanonical signaling pathways involving kinases such as p38, ERK1/2, and AKT^47^. To determine whether LILRBs interact with TGF-β receptors, we cotransfected HEK293T cells with *LILRB-Flag* and either *TGF-βRI-HA* or *TGF-βRII-HA*, followed by co-IP and western blot analysis. LILRBs interacted with both TGF-βRI and TGF-βRII, with a preferential association with TGF-βRI after normalization to the expression levels (Fig. 7a and Supplementary Fig. 9a). Notably, this interaction was partially inhibited by anti-LILRB2 blocking antibodies, suggesting a specific and regulatable association (Supplementary Fig. 9b). To determine whether this interaction occurs in *trans*, we independently transfected HEK293T cells with *LILRB2-Flag* or *TGF-βRI-HA* and coincubated them. Co-IP confirmed a trans interaction between LILRB2 and TGF-βRI, which was also partially inhibited by anti-LILRB2 blocking antibodies (Fig. 7b). This interaction was further validated in primary neutrophils, where Co-IP assays confirmed LILRB2–TGF-βRI binding (Supplementary Fig. 9c).

TGF-βRI contains an extracellular ligand-binding domain, a transmembrane region, and a juxtamembrane glycine-serine (GS) domain, followed by a serine/threonine kinase domain. The extracellular domain is essential for recruitment to the TGF-β–TGF-βRII complex^48^. To identify the TGF-βRI domain responsible for LILRB2 binding, we generated a series of truncated TGF-βRI mutants. Co-IP analysis revealed that the extracellular domain of TGF-βRI is required for its interaction with LILRB2 (Fig. 7c), suggesting that LILRB2 may facilitate the recruitment of TGF-βRI to TGF-βRII. To test this hypothesis, we cotransfected HEK293T cells with *TGF-βRI-Flag*, *TGF-βRII-HA*, and increasing concentrations of *LILRB2-Fc*. As expected, LILRB2 promoted the recruitment of TGF-βRI to TGF-βRII in a dose-dependent manner (Fig. 7d).

To further assess the role of LILRB2 in TGF-β signaling, we analyzed downstream signaling in THP-1 and THP-1-LILRB2 cells. Upon TGF-β1 stimulation, the phosphorylation of SMAD2 was significantly greater in THP-1-LILRB2 cells than in parental THP-1 cells, whereas the activation of noncanonical signaling pathways remained unchanged (Fig. 7e). Additionally, the interaction between LILRB2 and TGF-βRI was markedly increased following TGF-β1 stimulation (Fig. 7f), indicating that LILRB2 enhances SMAD2-dependent canonical TGF-β signaling.

Cystatin C has been reported to antagonize TGF-β signaling through interaction with TGF-βRII^49^. Regrettably, our analyses did not reveal a statistically significant interaction between cystatin C (either in its monomeric or oligomeric form) and TGF-β receptors (Supplementary Fig. 9d). To assess whether cystatin C affects TGF-β signaling, we generated *CST3*-knockout THP-1 cells (*THP-1 sgCST3*). Unexpectedly, SMAD2 phosphorylation was significantly greater in *THP-1 sgCST3* cells than in *THP-1 sgScramble* cells (Supplementary Fig. 9e), indicating that physiological cystatin C functions as a negative regulator of TGF-β signaling.

We next examined whether oligomeric cystatin C influences LILRB2–TGF-βRI interactions in *cis* and *trans*. HEK293T cells were treated with cystatin C-containing EX-CELL supernatant after either cotransfection or individual transfection with *LILRB2-Flag* and *TGF-βRI-HA*. Oligomeric cystatin C significantly enhanced both the *cis-* and *trans*-interactions between LILRB2 and TGF-βRI (Fig. 7g). Consistent with these findings, SMAD2/SMAD3 phosphorylation was markedly increased in primary neutrophils and monocytes following cystatin C^EX^ treatment (Fig. 7h). Notably, these increases were partially reversed upon LILRB2 blockade (Fig. 7h), indicating that cystatin C modulates TGF-β signaling in a LILRB2-dependent manner. Taken together, our results suggest that the cystatin C–LILRB2 axis supports the immunosuppressive capacity of myeloid cells through both the classical ITIM–SHP pathway and the enhancement of TGF-β signaling. Cystatin C facilitates LILRB2–TGF-βRI interactions, leading to increased SMAD2/SMAD3 activation, ultimately promoting a more immunosuppressive TME.

### Oligomeric transthyretin binds to the inhibitory receptors LILRB2 and LILRB5

Given the conserved cross-β structural core characteristic of various amyloid fibrils^50^, we hypothesized that the interaction between amyloid proteins and the LILRB2 or LILRB5 receptor may represent a broader phenomenon. To test this hypothesis, we assessed the activation potential of LILRB reporter cells in response to four well-characterized amyloid proteins: transthyretin (TTR), prion protein (PrP), serum amyloid A4 (SAA4), and α-synuclein. Consistent with our findings for cystatin C, plates coated with TTR and SAA4, but not PrP or α-synuclein, significantly activated LILRB2 and LILRB5 reporter cells (Supplementary Fig. 10). These results suggest that TTR and SAA4 are potential extracellular ligands for the inhibitory receptors LILRB2 and LILRB5.

**Fig. 8:**
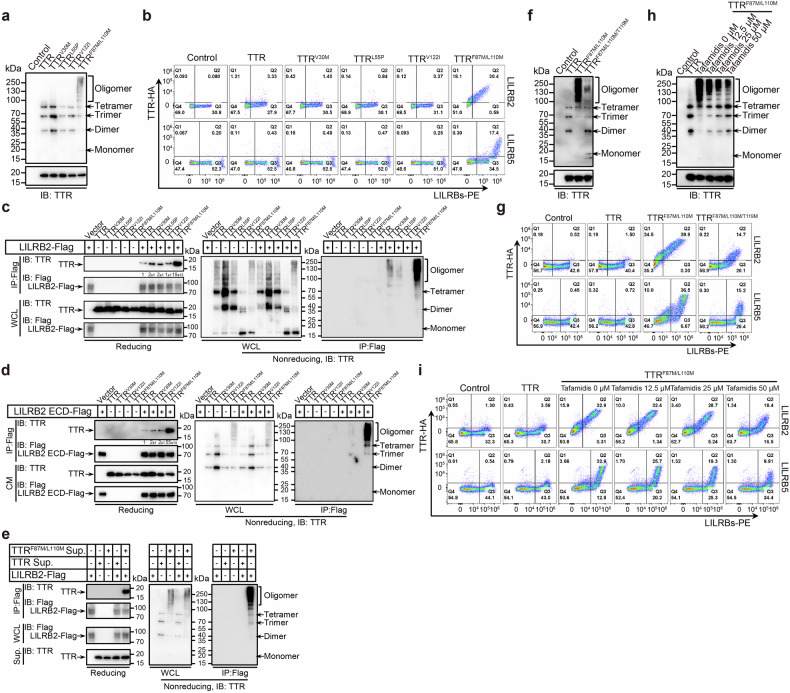
Oligomeric TTR binds to the LILRB2 and LILRB5 receptors. **a** Western blot analysis of WT or mutated transthyretin (TTR) in DMEM-conditioned medium from HEK293T cells expressing WT or mutated TTR via an anti-TTR monoclonal antibody under nonreducing (top) and reducing (bottom) conditions. **b** Flow cytometry analysis of WT or mutated TTR-HA in DMEM-conditioned medium binding to HEK293T cells expressing LILRB2 or LILRB5. **c** Co-IP assays revealed that TTR oligomers bind to LILRB2 in cotransfected HEK293T cells. Band intensities were quantified relative to the input and are presented as the means ± SD. *n* = 3 biological replicates. **d** Co-IP assays revealed that TTR oligomers bind to the ECD of LILRB2 in the CM of cotransfected HEK293T cells. Band intensities were quantified relative to the input and are presented as the means ± SD. *n* = 3 biological replicates. **e** Co-IP assays revealed that TTR oligomers bind to LILRB2 on the surface of HEK293T cells expressing LILRB2 following incubation with TTR-containing DMEM. **f** Western blot analysis of TTR^F87M/L110M/T119M^ in DMEM-conditioned medium from HEK293T cells expressing mutated TTR via an anti-TTR monoclonal antibody under nonreducing (top) and reducing (bottom) conditions. **g** Flow cytometry analysis of TTR^F87M/L110M/T119M^-HA in DMEM-conditioned medium bound to HEK293T cells expressing LILRB2 or LILRB5. **h** Western blot analysis of TTR^F87M/L110M^ in DMEM-conditioned medium after Tafamidis treatment with an anti-TTR monoclonal antibody under nonreducing (top) and reducing (bottom) conditions. **i** Flow cytometry analysis of Tafamidis-treated TTR^F87M/L110M^-HA in DMEM-conditioned medium binding to HEK293T cells expressing LILRB2 or LILRB5. See also Supplementary Fig. 10

We selected TTR as a representative amyloid protein to investigate its interaction with LILRB2 and LILRB5. TTR amyloid aggregation begins with the dissociation of native tetrameric TTR into monomers, a process driven by genetic mutations or age-related alterations. These monomers partially unfold and form amyloidogenic intermediates, which subsequently self-assemble into soluble oligomers and, eventually, amyloid aggregates^51^. To determine the binding form of TTR, we introduced three common amyloidogenic mutations—V30M, L55P, and V122I—along with a monomeric engineered F87M/L110M mutation^52,53^. Western blot analysis of DMEM-conditioned medium collected from HEK293T cells expressing these TTR variants revealed that the F87M/L110M mutation, but not the V30M, L55P, or V122I mutations, promoted dramatic TTR oligomerization (Fig. 8a). Flow cytometry analysis revealed that DMEM-conditioned medium containing TTR^F87M/L110M^ strongly bound to LILRB2- and LILRB5-transfected HEK293T cells, whereas the other TTR variants did not interact (Fig. 8b). Co-IP assays confirmed the molecular interaction between TTR and LILRB2. Cotransfection of *TTR* or *TTR* variants with *LILRB2-Flag* in HEK293T cells followed by co-IP and western blotting demonstrated that the oligomeric TTR^F87M/L110M^ variant exhibited a stronger interaction with LILRB2 than the WT and other TTR mutants did. Notably, the predominant binding form of TTR detected was its oligomeric state (Fig. 8c). To further confirm this interaction, we cotransfected HEK293T cells with *TTR* or *TTR* variants, and the extracellular domain (ECD) of LILRB2 fused to human IgG-Fc. Similar to our previous results, TTR^F87M/L110M^ oligomers strongly bound to the LILRB2 ECD (Fig. 8d). Additionally, incubation of LILRB2-transfected HEK293T cells with TTR^F87M/L110M^-containing DMEM further validated the cell–surface interaction between oligomeric TTR and LILRB2 (Fig. 8e).

TTR T119M is a nonpathogenic variant commonly found in the Portuguese population^54^. To assess whether the T119M mutation affects TTR oligomerization and its interaction with LILRB2 and LILRB5, we introduced the T119M mutation into TTR^F87M/L110M^. As expected, the T119M mutation significantly inhibited TTR^F87M/L110M^ oligomerization and reduced its binding affinity to LILRB2 and LILRB5 (Fig. 8f, g).

Tafamidis is an FDA-approved therapeutic agent designed to stabilize the native tetrameric structure of TTR and delay disease progression in transthyretin amyloidosis patients^55^. We investigated whether Tafamidis affects the interaction between TTR^F87M/L110M^ oligomers and LILRB2/LILRB5. Tafamidis treatment suppressed TTR^F87M/L110M^ oligomerization and reduced TTR^F87M/L110M^ binding to LILRB2 and LILRB5 in a dose-dependent manner (Fig. 8h, i). These findings demonstrate that oligomeric TTR interacts with the LILRB2 and LILRB5 receptors in a manner similar to that of cystatin C, suggesting a conserved mechanism of amyloid–LILRB interactions. Furthermore, the T119M mutation and Tafamidis treatment significantly reduced TTR oligomerization and binding to LILRB2 and LILRB5, highlighting potential therapeutic strategies to mitigate amyloid-driven immunosuppression.

## Discussion

Here, we identified the immune inhibitory receptors LILRB2 and LILRB5 as receptors for cystatin C, a cysteine cathepsin inhibitor, which defines a novel function for this secreted protein. We demonstrated that oligomeric cystatin C enhances the immunosuppressive activity of myeloid cells by engaging LILRB2/5 inhibitory receptors, thereby suppressing T-cell function and promoting cancer progression (Supplementary Fig. 11). Mechanistically, the immunosuppressive effects induced by the cystatin C–LILRB2 axis are mediated through the classical ITIM-SHP pathway and the upregulation of TGF-β signaling within these cells. Moreover, we identified strong interactions between transthyretin (TTR) oligomers—another well-characterized amyloid protein associated with systemic amyloidosis—and the LILRB2 and LILRB5 receptors. The discovery of additional ligands for LILRB2 and LILRB5 reinforces our view that LILRB family members mediate finely tuned immune functions that are regulated in multiple dimensions through interactions with ligands from diverse cellular and tissue sources^56^. This may reflect the relatively recent evolutionary emergence of LILRBs, which have the capacity to bind multiple ligands, enabling more precise signaling modulation and facilitating continued evolutionary refinement. Our results are also in line with previous reports that TGF-β signaling enhances the transcription of the *CST3* and *Src* genes while directly inducing Src kinase activation^49,57,58^. Importantly, our findings establish an unexpected molecular link between specific amyloid proteins, immunity, and cancer development, opening new avenues for therapeutic intervention.

Among the members of the cystatin superfamily, cystatin C is the most extensively studied in the context of tumor development and has been reported to function as both a tumor suppressor and promoter^12^. Physiologically, cystatin C adopts a characteristic fold comprising a five-stranded antiparallel β-sheet surrounding a central α-helix. Its secondary structure is as follows: (N)-β1-α-β2-L1 loop-β3-AS-β4-L2 loop-β5-(C). The native cystatin C monomer inhibits cysteine cathepsin through interactions mediated by N/L1/L2 binding motifs^59^. The tumor-suppressive role of cystatin C is attributed primarily to its ability to inhibit cysteine cathepsins—enzymes that promote invasion by degrading extracellular matrix (ECM) proteins and processing various growth factors and cytokines. This role has been extensively demonstrated in numerous in vitro and in vivo studies^60^. In contrast, cystatin C also has tumor-promoting functions, as evidenced by studies in B16-F10 melanoma metastasis models^61^ and in mammary tumor virus (MMTV) *polyoma middle T* (PyMT) transgenic mice^62^. Clinically, elevated cystatin C levels in body fluids are associated with poor prognosis in cancer patients^63^. To date, the tumor-supportive activity of cystatin C could be attributed to the complex functions of cathepsins in cancer and proteolysis-independent mechanisms. However, the precise molecular pathways involved remain unclear. Our findings provide mechanistic insight into this paradox: within the TME, cystatin C undergoes oligomerization, disrupting the conformation of the N/L1/L2 motif and abrogating its inhibitory activity against cysteine cathepsins. Simultaneously, oligomeric cystatin C binds to the inhibitory receptors LILRB2 and LILRB5 on myeloid cells, thereby increasing their immunosuppressive activity and contributing to tumor progression. Additionally, we observed moderate binding of cystatin C oligomers to the surface of B cells. Given the emerging role of B cells in both Alzheimer’s disease and cancer^64,65^, further studies are warranted to elucidate the regulatory impact of cystatin C oligomers on B-cell function.

A key question arises: what factors drive cystatin C oligomerization within the TME? We propose several possible mechanisms underlying this phenomenon. First, elevated cystatin C expression in cancer patients (Supplementary Fig. 1a) is the foundation for its oligomerization. Consistent with our findings, increased serum concentrations of cystatin C have been reported in patients with various cancers, such as melanoma and colorectal cancer, compared with healthy individuals^63^. However, the degree of cystatin C oligomer formation may not be directly proportional to high protein levels (Supplementary Fig. 1b). This discrepancy suggests that oligomerization is not solely dependent on expression levels. Elements such as local pH, oxidative stress, or specific protein‒protein interactions within the TME could significantly influence the oligomeric state of cystatin C (see below). Second, chemotherapy and radiotherapy may contribute to cystatin C oligomerization during cancer treatment. Research has shown that synchrotron radiation can induce domain swapping of human cystatin C^66^, a mechanism implicated in oligomerization and amyloid fibril formation. Additionally, glucocorticoids, which are commonly used as adjuvants during chemotherapy and radiotherapy, have been reported to increase cystatin C production under pathological conditions^9^. Glucocorticoids are also linked to increased cancer metastasis^67^. Importantly, cystatin C plays a critical role in enabling cancer cells to enter a radiation-tolerant state, allowing them to evade radiation and repopulate the tumor^11^. Therefore, caution is warranted when the body is exposed to high-energy radiation or when glucocorticoids are used to manage cancer-related complications. Third, biochemical characteristics of the TME, such as acidic pH, are associated with cystatin C oligomerization^68^. Studies on cystatin C have shown that acidic conditions disrupt three salt bridges between β2 and β3, facilitating the release of the N-terminal β1-α-β2-L1 loop motifs and subsequent domain swapping^69^. Given that both myeloid and tumor cells are major sources of cystatin C within the TME, prolonged exposure to an acidic environment likely contributes to its oligomerization. Finally, mutations in the *CST3* gene within tumor cells may lead to the production of unstable cystatin C monomers, promoting oligomerization. The TCGA pan-cancer atlas has identified 12 missense mutations in the *CST3* gene across different cancer types according to the cBioPortal platform, including R6H, V57F, F89L, R96Q, C109Y, R119M, K120I, A121E, F122I, A129S, Q133H, and S139L. While no studies have explicitly characterized the stability of cystatin C harboring these mutations, their critical position within the protein structure suggests that they may impact stability, at least to some extent. Drawing from therapeutic strategies used in other forms of amyloidosis—such as the TTR stabilizer Tafamidis, the RNA interference therapeutic Patisiran, and the monoclonal antibody CAEL-101, which targets the amyloid light chain (AL)—several potential approaches can be envisioned for targeting cystatin C: developing RNAi-based therapies to reduce the expression of mutant cystatin C; screening small molecules that stabilize the native conformation of cystatin C and prevent its misfolding; creating monoclonal antibodies that specifically recognize and facilitate the clearance of cystatin C amyloid deposits; and applying CRISPR-based gene editing to correct pathogenic mutations in familial cases, offering a precision medicine approach. While these strategies are promising, they face notable challenges—including the rarity of cystatin C-related disease, limited drug delivery across the blood–brain barrier and the absence of robust animal models. Nevertheless, targeting cystatin C represents a scientifically sound and potentially transformative direction for treating cancer and amyloid disorders.

TTR amyloidosis is associated with three clinical conditions: senile systemic amyloidosis (SSA), familial amyloid polyneuropathy (FAP), and familial amyloid cardiomyopathy (FAC). SSA is a late-onset disease characterized by the aggregation of wild-type (WT) TTR, leading to progressive cardiac muscle weakening. In contrast, FAP and FAC are hereditary disorders caused by the extracellular deposition of TTR amyloid fibrils in peripheral nerves and cardiac tissue, respectively. FAPs arise from various TTR mutations, including V30M and L55P, whereas the most prevalent mutation associated with FAC, V122I, is found in ~3.9% of the African–American population. Inflammation is a hallmark of several neurodegenerative disorders, including FAP^70^. Studies using a TTR V30M mouse model have shown that, following nerve injury, these mice exhibit reduced expression of cytokines and chemokines essential for recruiting immune cells, such as macrophages and neutrophils, both of which are critical for nerve regeneration^71^. Interestingly, asymptomatic FAP patients already exhibit elevated levels of the anti-inflammatory cytokines IL-33 and IL-10, suggesting that an anti-inflammatory response may precede fibril deposition^72^. The receptor for advanced glycation end products (RAGE) is a well-known mediator of inflammation and is recognized for interacting with soluble oligomers of various amyloid proteins, including TTR and SAA^73,74^. Through these interactions, RAGE contributes to chronic inflammation, driving the pathogenesis of neurodegenerative diseases and systemic amyloidosis. Like RAGE, our findings revealed that LILRB2 and LILRB5 can bind soluble oligomers of these amyloid proteins. These results suggest that further investigations are warranted to explore the functional significance of LILRB-mediated anti-inflammatory responses in the pathogenesis of amyloidosis.

To date, the relationship between amyloidosis and cancer remains poorly understood, with observed correlations largely limited to specific amyloidosis subtypes and certain cancers. For example, extensive epidemiological studies have established a bidirectional association between Parkinson’s disease and melanoma despite their contrasting pathological hallmarks—one characterized by neurodegeneration and the other by uncontrolled proliferation. Evidence suggests that Parkinson’s disease increases the risk of melanoma and vice versa^75^. Notably, an epidemiological study reported that ~50% of patients with both melanoma and parkinsonism have a primary melanoma on the head or neck, compared with only 6% of melanoma patients without parkinsonism^76^. Given that melanomas in these regions have a greater propensity to metastasize to the brain, this finding raises the intriguing possibility of a link between Parkinson’s disease and brain metastasis. More recently, a positive correlation between Alzheimer’s disease and melanoma brain metastasis has been identified. Research suggests that soluble Aβ oligomers secreted by melanoma cells drive an anti-inflammatory phenotype in astrocytes and microglia, promoting melanoma survival and proliferation in the brain parenchyma^77^. Our findings provide a potential mechanistic explanation for this phenomenon: amyloid proteins activate LILRB2 and LILRB5 inhibitory receptors on myeloid cells, establishing an immunosuppressive TME that supports tumor growth and metastasis. Moreover, cancer has been shown to contribute to amyloidosis progression. Approximately 10–15% of multiple myeloma patients develop amyloid light chain (AL) amyloidosis in the later stages of their disease^78^. In addition, renal cell carcinoma and Hodgkin lymphoma have been associated with the onset of serum amyloid A (AA) amyloidosis^79,80^. Understanding these molecular links is crucial for early diagnosis and personalized treatment strategies, as coexisting malignancies and amyloidosis can significantly impact patient outcomes. Further research is needed to delineate the mechanisms underlying the cancer–amyloidosis relationship and to develop effective therapeutic approaches for their comanagement.

## Materials and methods

### Patient samples

Blood and tissue samples from cancer patients were obtained through the University of Texas Southwestern (UTSW) Tissue Management Shared Resource with Institutional Review Board (IRB) approved protocol (STU 102010-051). The samples were distributed to the laboratory in a deidentified fashion, and the proposed study was not considered human research.

### Mice

C57BL/6J and NOD-SCID IL2Rγ-null-SGM3 (NSG-SGM3) mice (Jax #013062, which are NSG mice with transgenic expression of human SCF, GM-CSF, and IL-3 to enable better engraftment of human myeloid cells), were purchased from Jackson Laboratory and maintained at the animal core facility of UTSW. *Cystatin C*−*/−* mice (stock number: 047241-UCD) and *PIRB*−*/−* mice (PIRBTM, stock number: 030668-UCD) were obtained from the Mutant Mouse Resource & Research Center (MMRRC). Myeloid-specific (*LysM-Cre*) LILRB2 (LILRB2^KI^) or LILRB5 (LILRB5^KI^) transgenic mice were produced via CRISPR and backcrossed with WT C57BL/6J mice for at least six generations, as described previously^25,33^. The animal work described in this manuscript was approved and conducted under the supervision of the UT Southwestern Institutional Animal Care and Use Committee (IACUC) (protocol number: 2016-101657). For each experiment, the same sex- and age-matched (6–8 weeks) mice were used and randomly allocated to each group. For tumor size measurement, the experimenters were blinded to the treatment conditions of the mice. For the subcutaneous tumor model, the tumor size was calculated as (length × width × width)/2 mm^3^. The maximal tumor measurement permitted by UTSW IACUC is 2 cm in diameter or 2000 mm^3^ in volume. We complied with all relevant ethical regulations and used approved animal study protocols.

### Cell culture

HEK293T, B16-F10, CT-2A, and SK-MEL-5 cells were cultured in Dulbecco’s modified Eagle’s medium (DMEM) (Corning, cat. no. 10-013-CV) supplemented with 10% fetal bovine serum (FBS) (Thermo Fisher, cat. no. A5256701) at 37 °C in 5% CO_2_ and normal O_2_. EX-CELL^®^ 293 serum-free medium (Sigma, cat. no. 14571C) was used to collect oligomeric cystatin C from HEK293T cells expressing cystatin C 18 h post-transfection. DMEM was used to collect oligomeric TTR from HEK293T cells expressing TTR 18 h post-transfection. Human monocytic THP-1 (ATCC, TIB-202) and primary cells were cultured in Roswell Park Memorial Institute (RPMI) 1640 medium (Corning, cat. no. 10-040-CV) supplemented with 10% FBS at 37 °C in 5% CO_2_ and normal O_2_. All the cell lines were routinely tested via a mycoplasma contamination kit (R&D Systems, cat. no. CUL001B).

### Plasmids

Human *CST3* (Accession No. NM_000099.2) and mouse *CST3* (Accession No. NM_009976.3) cDNAs were cloned from plasmids obtained from SinoBiological (Cat. Nos. HG10439-G and MG50239-M, respectively). Human *TTR* cDNA (Accession No. NM_000371.3) was purchased from the UTSW Human Genetics Center. A Flag tag, HA tag, or 6×His tag was fused to the C-terminus of *CST3*, *TTR*, or their point-mutated variants. The resulting constructs were inserted into the pLVX lentiviral expression vector under the control of the EF1α promoter. To achieve cystatin C depletion, human or mouse *CST3* gRNA was cloned and inserted into the LentiCRISPR v2 vector (RRID: Addgene_52961). *LILRB2* (UniProt No. Q8N423), *LILRB2-ECD*, *LILRB5* (UniProt No. O75023), *LILRB5-ECD*, *PIRB* (UniProt No. P97484), and *gp49B* (UniProt No. Q64281) were cloned from synthesized cDNAs (Genewiz) and subcloned and inserted into the pLVX-M-Puro lentiviral expression vector (RRID: Addgene_125839) with either a C-terminal Flag tag or Fc tag. *LILRB2* domain-swapping constructs were generated by inserting an N-terminal HA tag following the signal peptide and replacing specific Ig-like domains with those from *LILRB1* (UniProt No. Q8NHL6). *LILRB5* truncation constructs were generated by inserting an N-terminal Flag tag after the signal peptide and deleting one or more Ig-like domains. *SHP1* (UniProt No. Q9BZQ2), *SHP2* (UniProt No. Q06124), and *Lyn* (UniProt No. P07948) were cloned from synthesized cDNA (Genewiz) and inserted into the pLVX-M-Puro vector (RRID: Addgene_125839). *Src* (Accession No. NM_005417.3), *TGF-βRI* (NM_004612.4), and *TGF-βRII* (NM_003242.5) cDNAs were obtained from the UTSW Human Genetics Center and subcloned and inserted into the same vector, with either an N-terminal HA tag following the signal peptide or a C-terminal Flag or HA tag. All the plasmids were sequence-verified via whole-plasmid sequencing (Eurofins Genomics).

### Quantification of circulating human cystatin C by ELISA

Circulating levels of human Cystatin C were quantified via a Quantikine ELISA Kit (R&D Systems, cat. no. DSCTC0) according to the manufacturer’s instructions. Briefly, standards, blanks, and 30-fold diluted serum samples were added to a 96-well microplate pre-coated with an anti-human cystatin C antibody. The plate was incubated at 4 °C for 3 h. After four washes with the provided wash buffer, a horseradish peroxidase (HRP)-conjugated detection antibody was added to each well, and the samples were incubated at 4 °C for 1 h. Following a final wash step, substrate solution was added to initiate color development, and the reaction was terminated with 2 N H_2_SO_4_. The absorbance was measured at 450 nm via a microplate reader.

### Dot blot

Equal volumes of serum were dotted onto nitrocellulose membranes. After the samples were blocked with 5% milk, the levels of human or mouse cystatin C were detected via an anti-cystatin C monoclonal antibody and an HRP-conjugated anti-rabbit secondary antibody. The levels of total amyloid oligomers were detected via an anti-oligomer (A11) polyclonal antibody and an HRP-conjugated anti-rabbit secondary antibody. Chemiluminescence was detected via a chemiluminescent substrate and chemiluminescent imaging. Albumin levels were determined with an anti-albumin antibody (Millipore, cat. no. SAB4200711), which served as an internal control.

### Flow cytometry

The cells from healthy donors, cancer patients, naïve mice, and tumor-bearing mice were washed with FACS buffer (PBS supplemented with 2% FBS and 1% penicillin/streptomycin) and then blocked with human IgG (Sigma, cat. no. I4506) or mouse IgG (Sigma, cat. no. I5381) for 20 min at 4 °C. The cells were subsequently stained with primary antibodies (Supplementary Table 3) for 30 min at 4 °C. To assess the degree of cystatin C or TTR binding, the indicated protein-containing supernatants were incubated with primary cells or HEK293T cells expressing LILRB2 or LILRB5 for 1 h at 4 °C prior to antibody staining. Propidium iodide (Sigma, cat. no. P4864) staining after primary antibody staining or zombie yellow (Biolegend, cat. no. 423104) staining before primary antibody staining was used to exclude dead cells from analysis. Intracellular staining of IFN-γ, perforin, granzyme or p-SMAD2/SMAD3 was performed via a Fixation/Permeabilization Kit (BD Biosciences, cat. no. 554714) according to the manufacturer’s protocol. Intracellular staining of p-STAT3 was performed with True-Phos Perm Buffer (BioLegend, 425401) according to the manufacturer’s protocol. Flow cytometry data were collected via FACS Melody or Cytek Northern Lights. Flow cytometry data were analyzed via FlowJo software.

### Western blotting

Whole cells were washed twice with ice-cold PBS and lysed on ice with freshly prepared ice-cold cell lysis buffer containing 50 mM Tris-HCl (pH 7.4), 150 mM NaCl, 1% NP-40 (w/v), 1 mM EDTA, 10% glycerol (w/v), a protease inhibitor cocktail (Sigma, cat. no. 11836153001) and a phosphatase inhibitor (Sigma, cat. no. 4906837001). Non-reduced samples were added with Laemmli sample buffer (Bio-Rad, cat. no. 1610737) and loaded on ExpressPlus™ PAGE (GenScript, cat. no. M42015) 4–20% gels without heating/boiling. In reduced samples, β-mercaptoethanol (5% final concentration) were added. Reduced samples were boiled at 95 °C for 10 min and separated on SDS–PAGE gels. After being transferred to nitrocellulose membranes, the membranes were blocked with 5% dry milk diluted in TBST. Proteins were detected with specific primary antibodies (Supplementary Table 4) and HRP-conjugated anti-mouse secondary antibodies (Jackson ImmunoResearch, cat. no. 115–035–003) or HRP-conjugated anti-rabbit secondary antibodies (Jackson ImmunoResearch, cat. no. 111–035–003). Chemiluminescence was detected via a chemiluminescent substrate (Millipore, cat. no. WBLUR0500) and chemiluminescent imaging (Bio-Rad, Chemidoc).

### Purification of oligomeric cystatin C

HEK293T cells were transfected with the *CST3* expression plasmid (see Plasmids) via PolyJet™ transfection reagent (SignaGen, cat. no. SL100688). After 18 h, the medium containing the PolyJet™/DNA complexes was replaced with EX-CELL® 293 serum-free medium (Sigma, cat. no. 14571C) for the collection of oligomeric cystatin C. Supernatants were harvested 48 h later and centrifuged to remove cell debris. For purification, HisPur™ Ni-NTA resin (Thermo Fisher, cat. no. 88222) was equilibrated with binding buffer and directly added to the filtered supernatant. The mixture was gently rotated overnight at 4 °C to allow binding. Then, the supernatant was loaded onto a gravity column to collect the resin. The resin was washed with binding buffer containing 20 mM imidazole, and the His-tagged protein was eluted with buffer containing 300 mM imidazole. The eluted fractions were dialyzed via a dialysis kit (Sigma, cat. no. PURX12015) for buffer exchange and then stored at −80 °C.

### Co-IP and pull-down

For in vivo Co-IP, LILRB-, PIRB- or TGF-βRI-Flag complexes in transfected HEK293T cells were immunoprecipitated with anti-FLAG^®^ M2 magnetic beads (Sigma, cat. no. M8823) per the manufacturer’s protocol; the LILRB2 ECD-Fc or LILRB5 ECD-Fc complexes in conditioned medium of cotransfected HEK293T cells were immunoprecipitated with Dynabeads Protein A beads (Invitrogen, cat. no. 10001D) per the manufacturer’s protocol. The phosphotyrosine complex in stable THP-1-LILRB2 or THP-1-LILRB5 cells was captured by a phosphotyrosine (4G10) monoclonal antibody (CST, cat. no. 96215) followed by immunoprecipitation with protein A Sepharose (Cytiva, cat. no. 17-0963-03); the TGF-βRI complex in THP-1-LILRB2 stable cells or primary neutrophils was captured by a TGF-βRI antibody (D-1) monoclonal antibody (Santa Cruz Biotechnology, cat. no. sc-518018), followed by immunoprecipitation with protein A Sepharose (Cytiva, cat. no. 17-0963-03). For in vitro pull-down, purified cystatin C^EX^-His was incubated with commercial LILRB2 ECD-Fc or LILRB5 ECD-Fc in PBS for 2 h followed by immunoprecipitation with Dynabeads™ His-Tag beads (Invitrogen, cat. no. 10103D) and western blotting.

### Cystatin C binding to LILRB2 and LILRB5 as measured by ELISA

Two-fold serial diluted cystatin C^EX^ protein was coated on a high-binding ELISA plate by incubation overnight at 4 °C. After blocking with 1% BSA in PBS, 10 μg/mL LILRB2 ECD- or LILRB5 ECD-Fc fusion protein was added, and the mixture was incubated for 2 h at room temperature. After washing with PBST, anti-human-IgG-Fc-HRP (Jackson ImmunoResearch, cat. no. 109-035-008) was added at a 1:1000 dilution and incubated for 1 h at room temperature. After washing with PBST, TMB substrate solution (BioLegend, cat. no. 421101) was added, and the mixture was incubated for 5 min before the addition of 2 N H_2_SO_4_ to stop the reaction. Optical density (OD) values were read at 450 nm.

### Octet binding kinetics

The kinetics of the binding of monomeric and oligomeric cystatin C to LILRB2 and to LILRB5 were analyzed via biolayer interferometry (Octet Red 384, FortéBio). Recombinant LILRB2-ECD-Fc (ACROBiosystems, cat. no. LI2-H5253) or LILRB5-ECD-Fc (R&D Systems, cat. no. 10410-T4) at a concentration of 100 nM was immobilized onto preequilibrated protein A biosensors (Sartorius, cat. no. 18-5010) in 1 × kinetics buffer (Sartorius, cat. no. 18-1105). The biosensors were subsequently dipped into varying concentrations of monomeric cystatin C^Sino^ (Sino Biological, cat. no. 10439-H08H; 156, 313, 625, 1250, 2500, 5000 nM) or oligomeric cystatin C^EX^ (156, 313, 625, 1250, 2500, 5000 nM) proteins. The association and dissociation phases were set to 600 and 1200 s, respectively. The kinetic curves were fitted to a 1:1 binding model via Octet System Data Analysis Software (FortéBio). Double referencing, which uses both the reference well and reference sensor, was used to account for nonspecific binding.

### Chimeric receptor reporter assay

LILRB chimeric receptor reporter cells were developed as described previously^23^. Fifty microliters (20 µg/ml) of commercial cystatin C^Sino^ (Sino Biological, cat. no. 10439-H08H) or purified cystatin C^EX^ were pre-coated onto 96-well plates at 37 °C for 3 h. After two washes with PBS, 1 × 10^5^ LILRB reporter cells were seeded in each well. When soluble proteins were present, equal amounts of recombinant proteins were mixed with the reporter cells before seeding. Moreover, the indicated isotype (10 μg/mL), anti-LILRB2 blocking (10 μg/mL) or anti-LILRB5 blocking antibodies were added to the culture medium. After culture for 18 h, the percentage of GFP^+^ reporter cells was measured via flow cytometry. The threshold of activation was 2 times greater than that of the negative control treatment.

### Measurement of endotoxin levels in cystatin C^EX^

Endotoxin levels of cystatin C^EX^ were quantified via the Pierce™ Chromogenic Endotoxin Quant Kit (Thermo Fisher, cat. no. A39553) according to the manufacturer’s instructions.

### In vitro differentiation of macrophages and dendritic cells from primary human CD14^+^ monocytes

Normal human CD14^+^ monocytes were isolated from the mononuclear cell fraction of normal peripheral blood via the AutoMACS Pro Separation System (Miltenyi Biotec). In brief, the buffy coat was purchased from the Interstate Blood Bank, and the mononuclear cell layer was separated by Ficoll Hypaque (GE Life Sciences, cat. no. 17144003) density gradient centrifugation. Mononuclear cells were treated with red blood cell lysis buffer to remove red blood cells and then incubated with anti-CD14 microbeads (Miltenyi Biotec, cat. no. 130–050–201) for 15 min at 4 °C. CD14-positive cells were then isolated via the positive selection program according to the manufacturer’s protocol. The protocol for M1 and M2 differentiation was conducted as previously described^31^. To develop M1 macrophages, 1 × 10^5^ CD14^+^ cells were cultured in RPMI 1640 medium supplemented with 10% FBS and 25 ng/ml GM-CSF (PeproTech, cat. no. 300-03) per well of a 96-well plate for 6 days. The medium was replaced with fresh medium every 3 days. To develop M2a macrophages, 1 × 10^5^ CD14^+^ cells were cultured in macrophage-SFM (Gibco, cat. no. 12065--074) supplemented with 100 ng/ml M-CSF (Sino Biological, cat. no. 11792-H08H) per well of a 96-well plate for 6 days. Fresh medium was replaced every 3 days, and 20 ng/ml IL-4 (Sino Biological, cat. no. 11846-HNAE) was added on day 3. Polarization started on day 6 with the medium used on day 3 supplemented with 100 ng/ml LPS (Sigma, cat. no. L4516) and lasted for another 24 h. The dendritic cell differentiation protocol was conducted as previously described^31^. A total of 1 × 10^5^ CD14^+^ cells were cultured in RPMI 1640 medium supplemented with 10% FBS, 50 ng/ml GM-CSF and 25 ng/ml IL-4 per well of a 96-well plate for 6 days. The medium was replaced with fresh medium every 3 days. The cells were stimulated on day 6 with RPMI 1640 medium supplemented with 10% FBS, 100 ng/ml LPS, and 1000 U/ml IFN-γ (PeproTech, cat. no. 300-02) for 48 h.

### In vitro polarization of neutrophils

Normal human CD15^+^ neutrophils were isolated via the AutoMACS Pro separation system from the granulocyte fraction of normal peripheral blood. In brief, the buffy coat was purchased from the Interstate Blood Bank, and the granulocyte layer was separated by Ficoll Hypaque density gradient centrifugation. Granulocytes were treated with red blood cell lysis buffer to remove red blood cells and then incubated with anti-CD15 microbeads (Miltenyi Biotec, cat. no. 130-094-530) for 15 min at 4 °C. CD15-positive cells were then isolated via the positive selection program according to the manufacturer’s protocol. Polarization of neutrophils toward an N1-like phenotype was conducted in a 96-well plate with 1 × 10^5^ CD14^+^ cells per well in RPMI 1640 medium supplemented with an N1 polarization cocktail containing 100 ng/ml LPS, 50 ng/ml IFN-γ, and 10,000 U/ml IFN-β (R&D Systems, cat. no. 8499-IF) for 1 day^81^. Since neutrophils have a short life span and undergo apoptosis within a few hours, the cultivation of neutrophils in the polarization experiments was carried out in the presence of 3 mM pan-caspase inhibitor Q-VD-Oph (Selleckchem, cat. no. S7311) to inhibit spontaneous neutrophil apoptosis and, consequently, increase their lifespan^82^.

### Neutrophil endocytosis assay

Normal human CD15^+^ neutrophils were isolated as described above. Q-VD-Oph (Selleckchem, cat. no. S7311) was used to inhibit spontaneous apoptosis of neutrophils. Neutrophils were seeded in 96-well plates at a density of 1 × 10⁵ cells per well and cultured in serum-free RPMI 1640 medium under the indicated treatment conditions for 12 h. Following treatment, 0.1 mg/mL FITC-conjugated dextran (MCE, cat. no. HY-128868A) was added to each well. The cells were then incubated at 37 °C for 45 min in the dark to allow endocytosis. To control for nonspecific binding and passive uptake, a parallel group was incubated with FITC-dextran at 4 °C. After incubation, the cells were washed three times with cold PBS to remove uninternalized FITC-dextran. The fluorescence intensity was measured via flow cytometry, and the mean fluorescence intensity (MFI) values were compared between the groups to assess the relative endocytic activity.

### Neutrophil migration assay

Human CD15⁺ neutrophils were isolated as described above. Q-VD-Oph (Selleckchem, cat. no. S7311) was used to inhibit spontaneous apoptosis of neutrophils. Neutrophils were pretreated with either an isotype control or anti-LILRB2 blocking antibody in serum-free RPMI 1640 medium for 4 h at 37 °C. Following treatment, 700 μL of serum-free RPMI 1640 medium was added to the lower chamber of a 24-well plate. Transwell inserts (Corning, cat. no. CLS3422) were carefully placed into each well. A total of 100 μL of neutrophil suspension (2 × 10⁶ cells) was added to the upper chamber of each insert. The cells were incubated at 37 °C in a humidified 5% CO₂ incubator for 17 h. After incubation, the transwell inserts were gently removed without disturbing the lower chamber. The number of migrated neutrophils in the lower chamber was quantified by flow cytometry.

### Cocultures of monocytes and autologous T cells

Monocytes and T cells were isolated from the mononuclear cell fraction of healthy buffy coats via anti-CD14 microbeads and anti-CD3 microbeads (Miltenyi Biotec, cat. no. 130-097-043), respectively. Monocytes were cocultured with carboxyfluorescein diacetate succinimidyl ester (CFSE)-stained autologous T cells (monocytes:T cells = 1:1) for 5 days and treated with the cystatin C^EX^ protein in the presence or absence of anti-LILRB2 (10 μg/ml) on day 0. A total of 12.5 μl/ml of ImmunoCult Human CD3/CD28 T-Cell Activator (StemCell, cat. no. 10971) was used to activate CD3^+^ T cells.

### Cocultures of MDSCs and autologous T cells

Coculture of MDSCs and autologous T cells was conducted as described previously^31,32^. MDSCs and T cells were isolated from the mononuclear cell fraction of the peripheral blood of the indicated cancer patients via anti-CD14 microbeads and anti-CD3 microbeads, respectively. MDSCs were cocultured with CFSE-stained autologous T cells (MDSCs:T cells = 2:1) for 5 days and treated with the cystatin C^EX^ protein in the presence or absence of anti-LILRB2 (10 μg/ml) on day 0. A total of 12.5 μl/ml of ImmunoCult Human CD3/CD28 T-Cell Activator was used to activate CD3^+^ T cells.

### IFN-γ ELISA

Conditioned media was collected from the cocultured system of MDSCs and autologous T cells on day 5. The samples were analyzed for IFN-γ secretion via a human IFN-γ ELISA Kit (BioLegend, cat. no. 430104) according to the manufacturer’s protocol.

### Human MDSC culture

MDSC culture was conducted as previously described^31,32^. Briefly, MDSCs isolated from the indicated cancer patients were cultured for 2 days and treated with the cystatin C^EX^ protein in the presence or absence of anti-LILRB2 (10 μg/ml) on day 0. The percentage of live MDSCs (propidium iodide-negative) and the surface expression of CD14 and CD163 were then analyzed via flow cytometry.

### Establishment of CST3 knockout and overexpression cell lines through lentivirus infection

For the *CST3* knockout cell lines, lentiCRISPR V2 plasmids encoding either a guide RNA (gRNA) targeting the coding region of the human or mouse *CST3* gene or a nontargeting (scramble) gRNA (Supplementary Table 5) were mixed with psPAX2 (Addgene, cat. no. 12260) and pMD2G (Addgene, cat. no. 12259) at a 4:3:1 ratio and transfected into HEK293T cells via Polyjet (SignaGen, cat. no. SL100688). After 48 h, the lentiviral supernatant was syringe-filtered through a 0.45 μm filter and centrifuged onto B16-F10 or THP-1 cells in the presence of 8 μg/mL polybrene (Sigma, cat. no. H9268). The virus-containing supernatant was removed after another 48 h, and monoclonal B16-F10 or THP-1 cells were selected with 5 μg/mL puromycin (Sigma, cat. no. P7255). For the *CST3*-overexpressing cell lines, *CST3* cDNA was cloned from commercial plasmids (Sino Biological, cat. no. HG10439-G) with a C-terminal FLAG tag and inserted into the pLVX lentiviral expression vector under the control of the *Ef1α* promoter. Lentiviral *CST3* supernatant was generated as described above and centrifuged onto B16-F10, CT-2A, or SK-MEL-5 cells in the presence of 8 μg/mL polybrene. After 48 h, B16-F10, CT-2A, or SK-MEL-5 cells were selected with 5 μg/mL puromycin.

### Tumor experiments in cystatin C−*/−* mice

A total of 5 × 10^5^ *B16-F10 sgScramble* or *B16-F10 sgCST3* melanoma cells were injected subcutaneously into the right flanks of WT and *cystatin C*−*/−* (C57BL/6J background) mice. Seven days later, the subcutaneous tumors were measured every two days via a digital caliper. The mice were euthanized on day 15. Tumor phenotypes were recorded, and tumor weights were measured. Spleens were harvested to isolate mouse MDSCs (CD11b^+^Gr1^+^) via the EasySep Mouse MDSC Isolation Kit (StemCell, cat. no. 19867).

### RT‒PCR

Total RNA was extracted via RNeasy Mini Kits (QIAGEN, cat. no. 74106). Each RNA sample (1 μg) was subjected to reverse transcription with HiScript IV RT SuperMix (Vazyme, cat. no. R423). Quantitative real-time PCR assays were performed in a QuantStudio^TM^ 6 Pro Real-Time PCR System (Applied Biosystems; cat. no. A43180) using Taq Pro Universal SYBR qPCR Master Mix (Vazyme, cat. no. Q712). The results were normalized according to the expression of *GAPDH* mRNA. After normalization, the expression of each target gene was calculated via the comparative threshold cycle (CT) method. The primers used for qPCR are shown in Supplementary Table 6.

### Tumor experiments in LILRB2^KI^ or LILRB5^KI^ mice

A total of 5 × 10^5^ B16-F10-vector or B16-F10-cystatin C melanoma cells were injected subcutaneously into the right flanks of WT and LILRB2^KI^ or LILRB5^KI^ (C57BL/6J background) mice. Seven days later, the subcutaneous tumors were measured every two days via a digital caliper. The mice were euthanized on day 15. For the indicated antibody experiments, the mice were randomized into two groups on the basis of tumor size, followed by intraperitoneal injection of 200 μg of isotype or anti-LILRB2 at days 4, 7, and 10. The subcutaneous tumors were measured every two days via a digital caliper beginning on day 7. The mice were euthanized on day 13. For the CT-2A-related tumor model, 2 × 10^6^ CT-2A-vector or CT-2A-cystatin C glioma cells were injected subcutaneously into the right flanks of WT and LILRB2^KI^ (C57BL/6J background) mice. Seven days later, the subcutaneous tumors were measured every five days via a digital caliper. The mice were euthanized on day 34.

### Tumor experiments with NSG-SGM3 and humanized mice

A total of 1 × 10^6^ SK-MEL-5-vector or SK-MEL-5-cystatin C human melanoma cancer cells were injected subcutaneously into the right flanks of NSG-SGM3 mice. Seven days later, the subcutaneous tumors were measured every seven days via a digital caliper. The mice were euthanized on day 49. For the humanized mouse model, 2 × 10^4^ human cord blood CD34^+^ cells (STEMCELL, cat. no. #70008) were transplanted into 2.5 Gy sublethally irradiated NSG-SGM3 mice as previously described^23,31^. To ensure consistent levels of human immune cell engraftment, only mice with ≥25% human CD45⁺ chimerism (with human myeloid and T cells) in the peripheral blood, as determined by flow cytometry 6 weeks post-transplantation, were included in the study. Mice meeting these criteria were stratified on the basis of the percentage of human CD45⁺ cells and then randomly assigned to experimental groups via a stratified randomization strategy to minimize variability in human immune cell reconstitution across groups. We then subcutaneously implanted 1 × 10^6^ SK-MEL-5-vector or SK-MEL-5-cystatin C human melanoma cancer cells into CD34^+^ humanized mice at week 8 in their right flanks. Seven days later, the subcutaneous tumors were measured every seven days via a digital caliper. For the indicated antibody experiments, the mice were randomized into two groups when the SK-MEL-5-cystatin C tumor size reached 100–120 mm^3^, followed by the intraperitoneal injection of 200 μg of isotype or anti-LILRB2 blocking antibody every 7 days thereafter.

### The Cancer Genome Atlas analyses

Patient data were obtained from The Cancer Genome Atlas (TCGA) via the GEPIA 2 Web Browser with the inclusion of all patients in each cancer subset. The *CST3* mRNA expression levels were determined by RNA-seq and divided into high or low groups according to the quartile cutoff of each subtype. Overall survival or disease-free survival was analyzed on the basis of *CST3* expression and corresponding patient survival data. Statistical significance was calculated via the log-rank (Mantel‒Cox) test.

### Statistical analysis

Statistical analyses were performed via GraphPad Prism 10 software. Statistical significance for two-sample comparisons was calculated via two-tailed Student’s *t* test. Statistical significance for multiple-sample comparisons was calculated by one-way ANOVA. Statistical significance for survival was calculated by the log-rank test. Multivariate analysis of TCGA data was performed via Cox regression. Differences were considered statistically significant if *P* < 0.05. Exact *P* values are shown.

## Supplementary information

SUPPLEMENTAL MATERIAL

Original and uncropped images of Western blots

## Data Availability

All the data needed to evaluate the conclusions are presented in the article and the Supplementary Materials.
