# Supplementary material for: Oligomeric cystatin C supports the immunosuppressive activity of myeloid cells through interaction with inhibitory receptors
Source: Signal Transduct Target Ther. 2025 Nov 14;10:368. doi: 10.1038/s41392-025-02462-x (PMC12615798; doi:10.1038/s41392-025-02462-x)
Supplement: Supplementary file 1 — SUPPLEMENTAL MATERIAL [file 41392_2025_2462_MOESM1_ESM.docx]

Supplementary Materials for

**Oligomeric** **cystatin C supports the immunosuppressive activity of myeloid cells through interaction with inhibitory receptors**

Chengcheng Zhang^1^, Yubo He^1^, Xiaoye Liu^1^, Jingjing Xie^1^, Meng Fang^1^, Xing Yang^1^, Ryan Huang^1^, Qi Lou^1^, Bufan Li^1^, Ankit Gupta^2^, Cheryl Lewis^3^, Marc I Diamond^2,4^, Ningyan Zhang^5^, Zhiq­­iang An^5^, Cheng Cheng Zhang^1^*

**Correspondence to:** **Alec.Zhang@UTSouthwestern.edu**

**The PDF file includes:**

Figures S1 to S11

Tables S1 to S6

**Supplementary Figures**


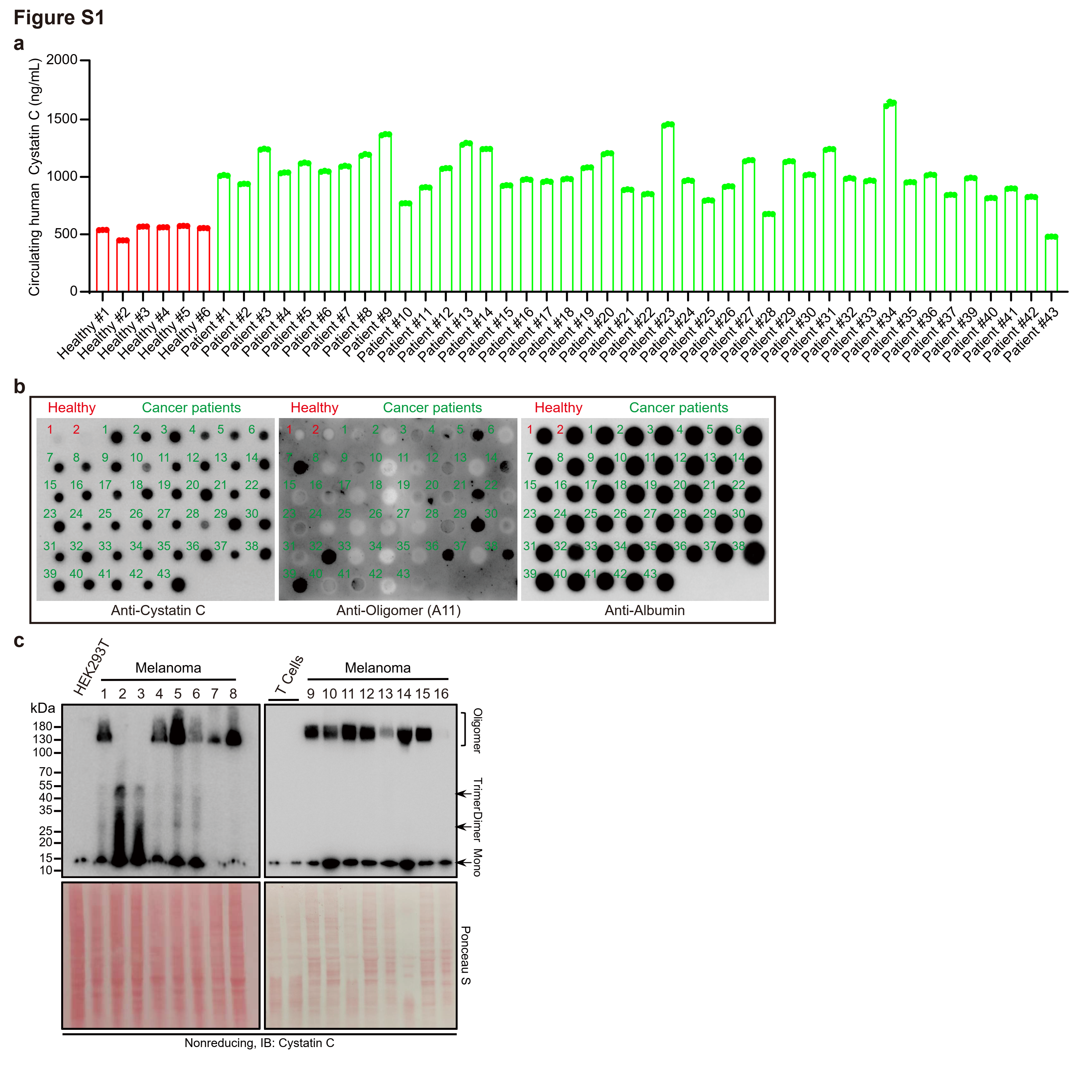


**Fig. S1 Characterization of the cystatin C levels and oligomerization in cancer patient.**

1. ELISA analysis of circulating cystatin C levels in the serum of healthy donors and cancer patients.
2. Dot blot analysis of cystatin C and total amyloid oligomer in the serum of healthy donors and cancer patients using anti-cystatin C monoclonal antibody and anti-Oligomer (A11) polyclonal antibody. Albumin levels served as the internal control.
3. Top: Western blot analysis of cystatin C oligomerization in melanoma tumor tissues using anti-cystatin C monoclonal antibody under nonreducing conditions. Bottom: The membrane stained by Ponceau S represented the total protein in each sample and served as the loading control.


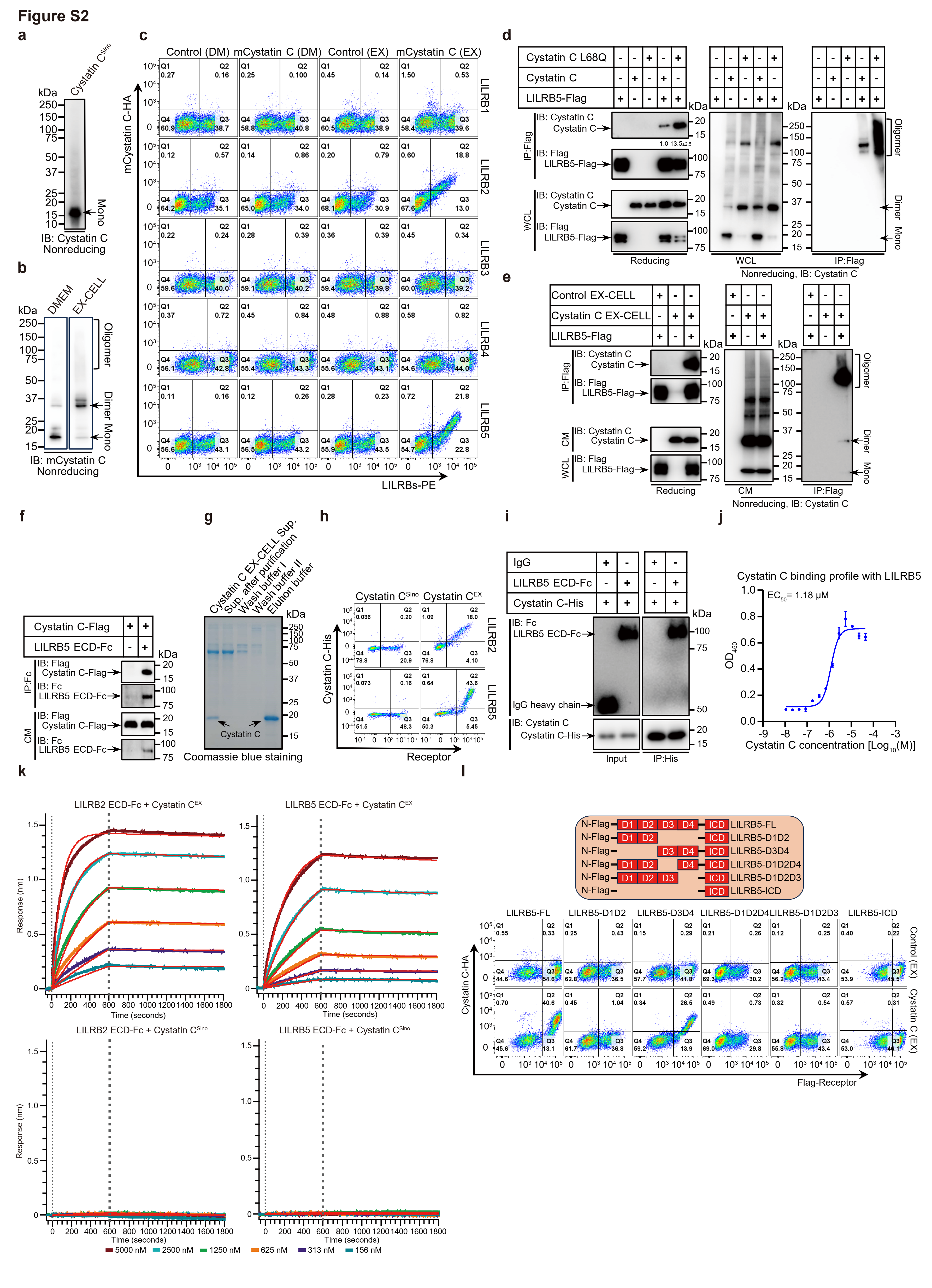


**Fig. S2 Characterization of the cystatin C oligomer-LILRB5 interaction, related to Fig. 1.**

1. Western blot analysis of commercial cystatin C recombinant protein using anti-cystatin C monoclonal antibody under nonreducing conditions.
2. Western blot analysis of mouse cystatin C in DMEM- or EX-CELL-conditioned medium from HEK293T cells expressing mouse cystatin C using anti-cystatin C monoclonal antibody under nonreducing conditions.
3. Flow cytometry analysis of mouse cystatin C-HA in DMEM- or EX-CELL-conditioned medium binding to HEK293T cells expressing LILRBs.
4. Co-IP assay showed that cystatin C oligomers bind to LILRB5 in cotransfected HEK293T cells. Band intensities were quantified relative to input and are presented as means ± SD. *n* = 3 biological replicates.
5. Co-IP assay showed that cystatin C oligomers bind to LILRB5 on the surface of HEK293T cells expressing LILRB5 following incubation with cystatin C-containing EX-CELL supernatant.
6. Co-IP assay showed that cystatin C binds to the ECD of LILRB5 in EX-CELL-conditioned medium (CM) from cotransfected HEK293T cells.
7. Purification of cystatin C-His protein from the EX-CELL-conditioned medium of HEK293T cells expressing cystatin C.
8. Flow cytometry analysis of cystatin C^Sino^ or cystatin C^EX^ binding to HEK293T cells expressing LILRB2 or LILRB5.
9. Pull-down assay showed that cystatin C^EX^ directly binds to LILRB5 ECD.
10. Titration curves of cystatin C^EX^ binding to LILRB5 ECD-Fc measured by ELISA. Data are presented as means ± SD.
11. Biolayer interferometry responses as a function of time of incubation of 100 nM LILRB2 ECD-Fc or LILRB5 ECD-Fc protein immobilized on protein A biosensors and dipped in various concentrations (156–5000 nM) of cystatin C^EX^ or cystatin C^Sino^.
12. Top: Schematic diagram showing the design of LILRB5 truncated mutants. Bottom: Flow cytometry analysis of cystatin C-HA in EX-CELL-conditioned medium binding to HEK293T cells expressing truncated LILRB5.


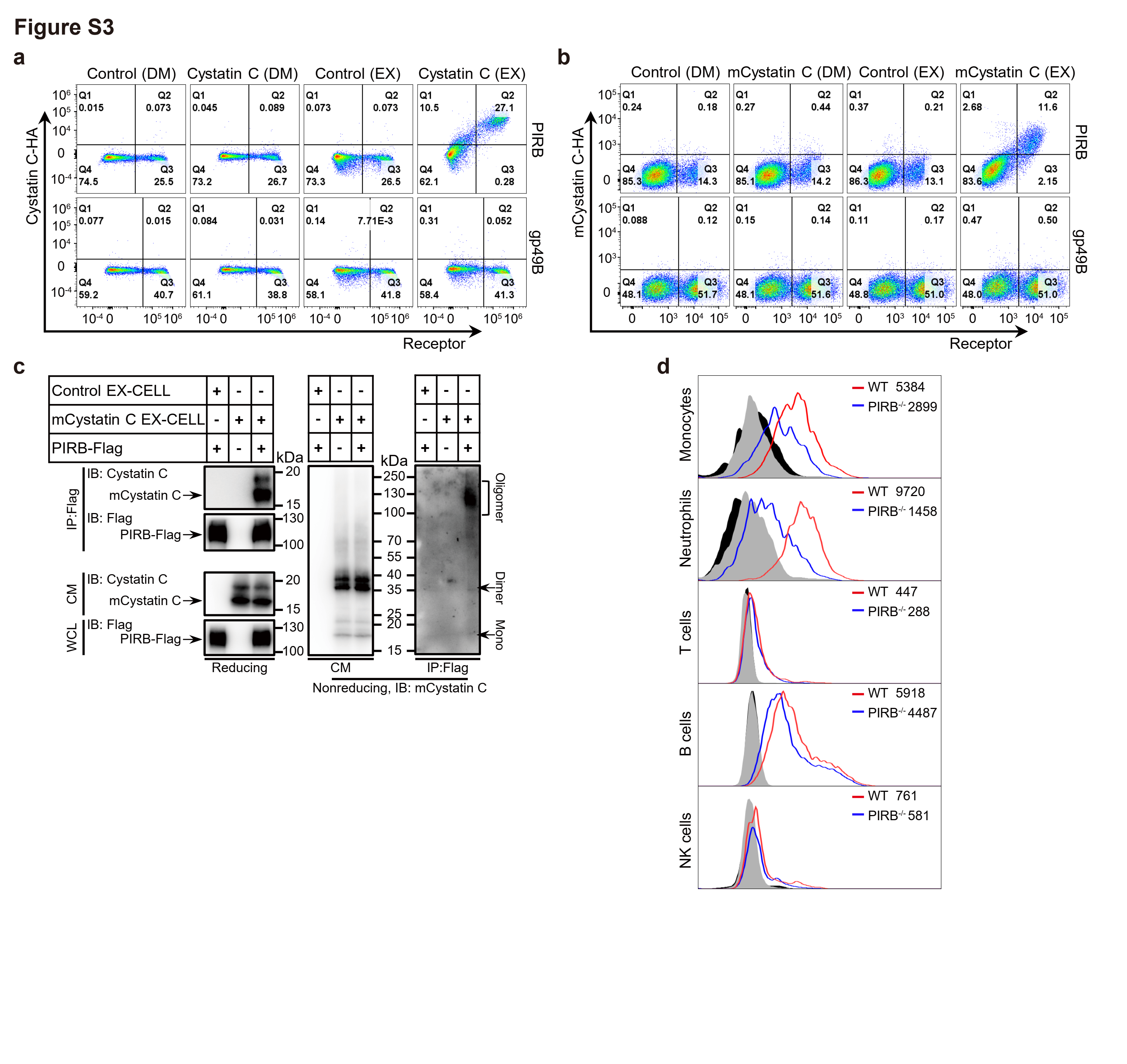


**Fig. S3 Characterization of the cystatin C oligomer-PIRB interaction, related to Fig. 1.**

1. Flow cytometry analysis of cystatin C-HA in DMEM- or EX-CELL-conditioned medium binding to HEK293T cells expressing PIRB or gp49B.
2. Flow cytometry analysis of mouse cystatin C-HA in DMEM- or EX-CELL-conditioned medium binding to HEK293T cells expressing PIRB or gp49B.
3. Co-IP assay showed that mouse cystatin C oligomers bind to PIRB on the surface of HEK293T cells expressing PIRB following incubation with mouse cystatin C-containing EX-CELL supernatant.
4. Representative flow cytometry histograms showing the binding of mouse cystatin C-HA in EX-CELL-conditioned medium to various primary immune cell populations from the peripheral blood of WT (red line) or *PIRB*−/− (blue line) mice. Gray-filled and black-filled histogram data are FMO controls for staining from WT or *PIRB*−/− mice, respectively. MFIs are indicated.


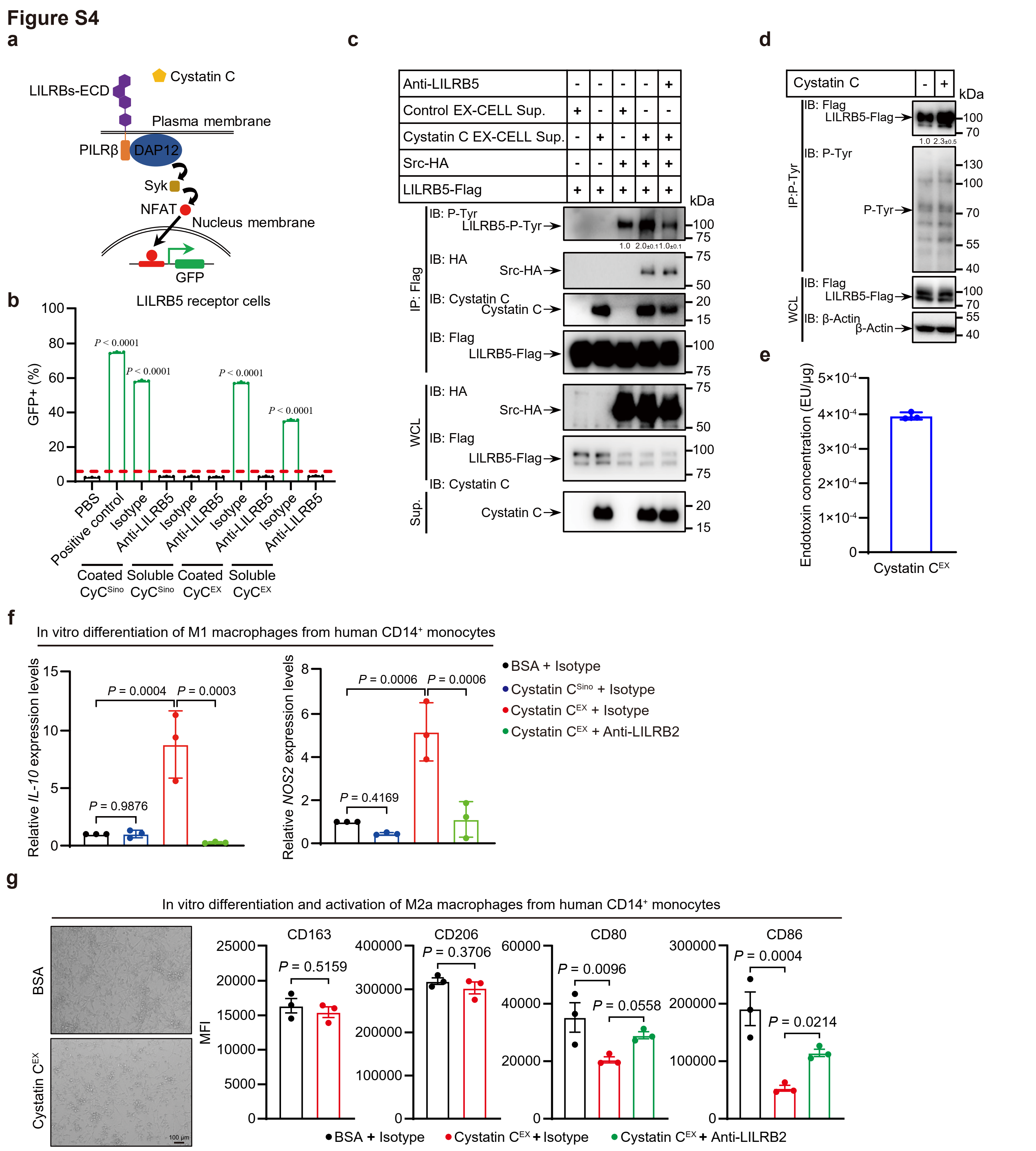


**Fig. S4 Cystatin C oligomers activate LILRB5 inhibitory receptor, related to Fig. 2.**

1. Schematic diagram of the chimeric LILRBs receptor reporter system.
2. Percentage of LILRB5 reporter cells activated on plates coated with or exposed to soluble commercial cystatin C^Sino^ (20 μg/mL) or cystatin C^EX^ (20 μg/mL) in the presence or absence of anti-LILRB5 (20 μg/mL) blocking antibody. *n* = 3 biological replicates. Data are presented as means ± SD. The threshold of activation is defined as twice that of the negative control treatment. *P* values were determined by one-way ANOVA with Dunnett’s multiple comparisons test.
3. Co-IP analysis of LILRB5-specific phosphotyrosine (P-Tyr) in HEK293T cells cotransfected with LILRB5-Flag and Src-HA plasmids, with or without treatment of anti-LILRB5 antibody, followed by incubation with cystatin C EX-CELL-conditioned medium. Band intensities were quantified relative to input and are presented as means ± SD. *n* = 3 biological replicates.
4. Co-IP analysis of LILRB5-specific P-Tyr in THP-1-LILRB5 stable cells after a 10-minutes incubation on plates coated with cystatin C (20 μg/mL). β-actin served as the internal control. Decimals, normalize to input. Band intensities were quantified relative to input and are presented as means ± SD. *n* = 3 biological replicates.
5. Endotoxin level of cystatin C^EX^ protein. Data are presented as means ± SD. *n* = 3 biological replicates.
6. RT-PCR of IL-10 and NOS2 expression in CD14^+^ monocytes-derived M1 macrophages. Data are presented as means ± SD. *n* = 3 biological replicates. *P* values were determined by one-way ANOVA with Holm-Sidak’s multiple comparisons test.
7. M2a macrophages were *in vitro* differentiated from CD14^+^ monocytes isolated from fresh human PBMCs. Cystatin C^EX^ and anti-LILRB2 antibodies were included in the differentiation from day 0. Left: Cells morphology on day 6, Scale bar = 100 µm. Right: MFIs of CD163, CD206, CD80, and CD86 were measured on day 7 using flow cytometry, when was 1 day after polarization. *n* = 3 biological replicates. Data are presented as means ± SD. *P* values were determined by two-tailed Student’s *t* test or one-way ANOVA with Holm-Sidak’s multiple comparisons test.


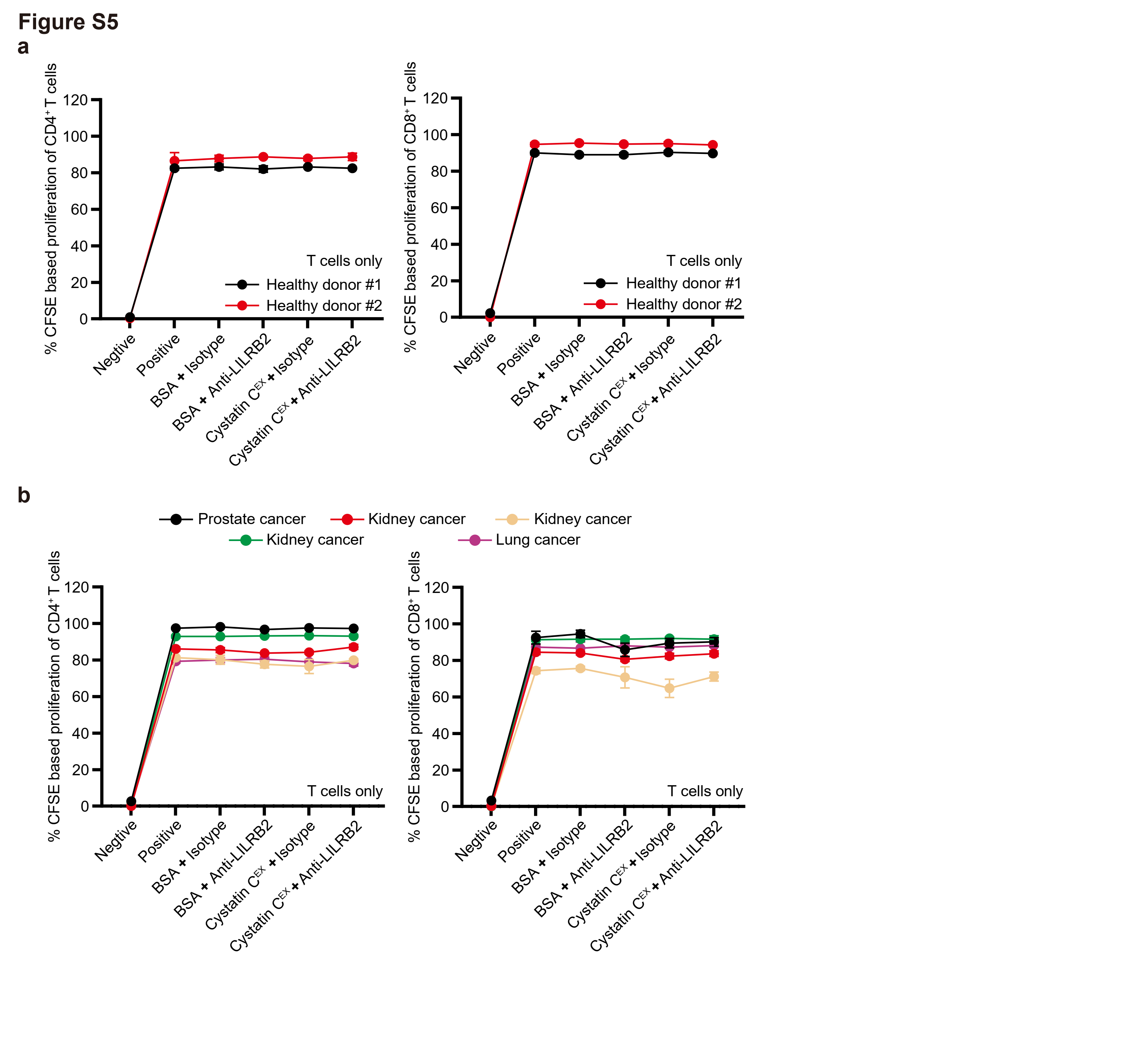


**Fig. S5 Effects of cystatin C oligomer on T cell** **proliferation, related to Fig. 3.**

1. Percentages of proliferative CD4^+^ and CD8^+^ T cells from healthy donors under the indicated treatment conditions.
2. Percentages of proliferative CD4^+^ and CD8^+^ T cells from cancer patients under the indicated treatment conditions.


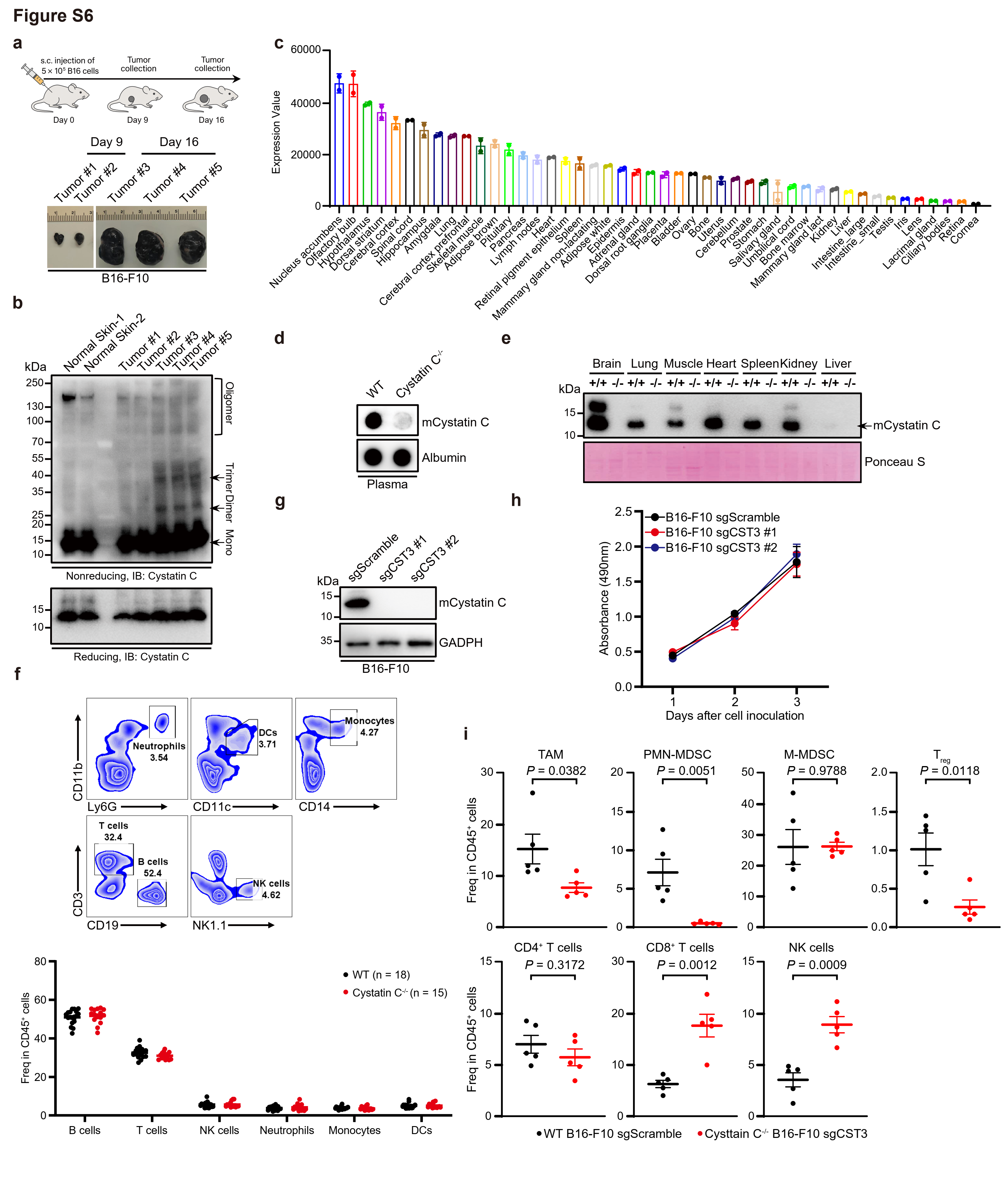


**Fig. S6 Characterization of cystatin C−/− mice and *CST3*-knockout cell line, related to Fig. 4.**

1. Representative photographs of B16-F10 tumors collected on days 9 and 16 from WT mice.
2. Western blot analysis of mouse cystatin C in normal skin and B16-F10 melanoma at different stages using anti-cystatin C monoclonal antibody under nonreducing conditions (Top) and reducing conditions (Bottom).
3. *CST3* mRNA expression in mouse tissues from BioGPS microarray database.
4. Dot blot analysis of mouse cystatin C levels in the plasma of WT or cystatin C−/− mice. Albumin levels served as the internal control.
5. Top: Western blot analysis of mouse cystatin C in the brain, lung, muscle, heart, spleen, kidney, and liver of WT and cystatin C−/− mice using anti-cystatin C monoclonal antibody. Bottom: The membrane stained by Ponceau S represented the total protein in each sample and served as the loading control.
6. Top: Gating strategy to analyze immune cell populations in peripheral blood. Bottom: Percentage of indicated immune cells in peripheral blood from WT (*n* = 18) and cystatin C−/− (*n* = 15) mice as measured by flow cytometry.
7. Western blot analysis of mouse cystatin C in monoclonal cell populations derived from B16-F10 cells transfected with either scrambled control RNA or guide RNAs (gRNAs) targeting the *CST3* gene locus. GADPH served as the internal control.
8. MTS assay showing the proliferation of *B16-F10 sgScramble* and *B16-F10 sgCST3* cells. Data are presented as means ± SD.
9. Percentages of the indicated immune cell types in *B16-F10 sgScramble* tumors from WT mice (*n* = 5) and *B16-F10 sgCST3* tumors from cystatin C*−/−* mice (*n* = 5) as measured by flow cytometry. *P* values were determined by two-tailed Student’s *t* test.


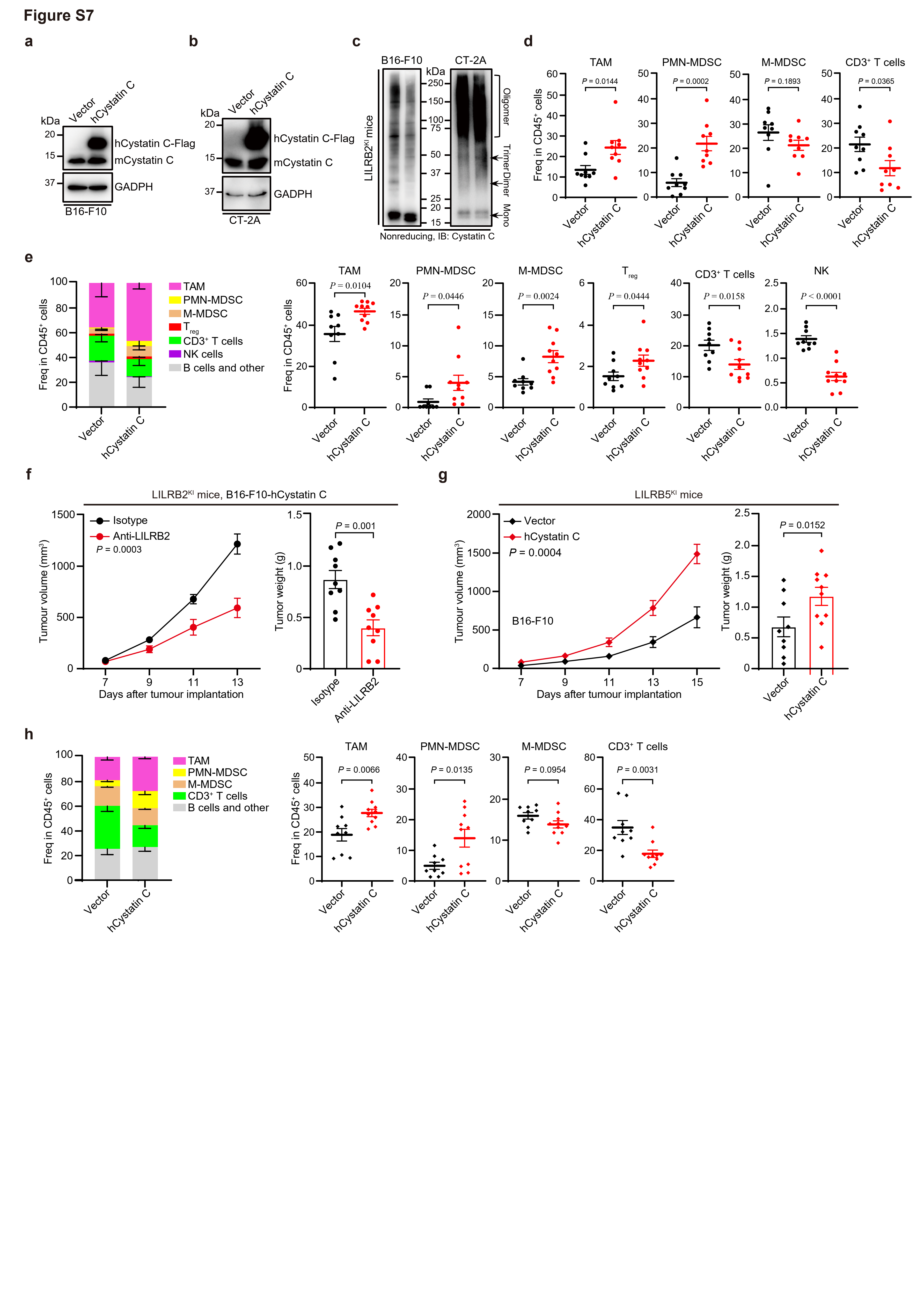


**Fig. S7 Forced expression of *CST3* promotes tumor growth in LILRB5^KI^ mice, related to Fig. 5.**

**(a, b)** Western blot analysis of human cystatin C in B16-F10 (A) or CT-2A (B) cells transfected with either vector plasmid or *hCST3*-*Flag* plasmid. GADPH served as the internal control.

1. Western blot analysis of human cystatin C within tumor microenvironment of *B16-F10-hcystatin C* and *CT-2A-hcystatin C* models in LILRB2^KI^ mice using anti-cystatin C monoclonal antibody under nonreducing conditions.
2. Percentage of indicated immune cell types in the *B16-F10-vector* or *B16-F10-hcystatin C* tumors of LILRB2^KI^ mice as measured by flow cytometry. Data are presented as means ± SEM. *P* values were determined by two-tailed Student’s *t* test.
3. Percentage of indicated immune cell types in the *CT-2A-vector* or *CT-2A-hcystatin C* tumors of LILRB2^KI^ mice as measured by flow cytometry. Data are presented as means ± SEM. *P* values were determined by two-tailed Student’s *t* test.
4. LILRB2^KI^ mice were inoculated subcutaneously with *B16-F10-hcystatin C* cells at day 0 and treated with isotype or anti-LILRB2 monoclonal antibody every 3 days from day 3 to day 12. Tumor growth curve and tumor weight in each group (*n* = 9) were shown. Data are presented as means ± SEM. *P* values were determined by two-tailed Student’s *t* test.
5. LILRB5^KI^ mice were inoculated subcutaneously with *B16-F10-vector* or *B16-F10-hcystatin C* cells. Tumor growth curve and tumor weight in each group (*n* = 9 or 10) were shown. Data are presented as means ± SEM. *P* values were determined by two-tailed Student’s *t* test.
6. Percentage of indicated immune cell types in the *B16-F10-vector* or *B16-F10-hcystatin C* tumors of LILRB5^KI^ mice as measured by flow cytometry. Data are presented as means ± SEM. *P* values were determined by two-tailed Student’s *t* test.


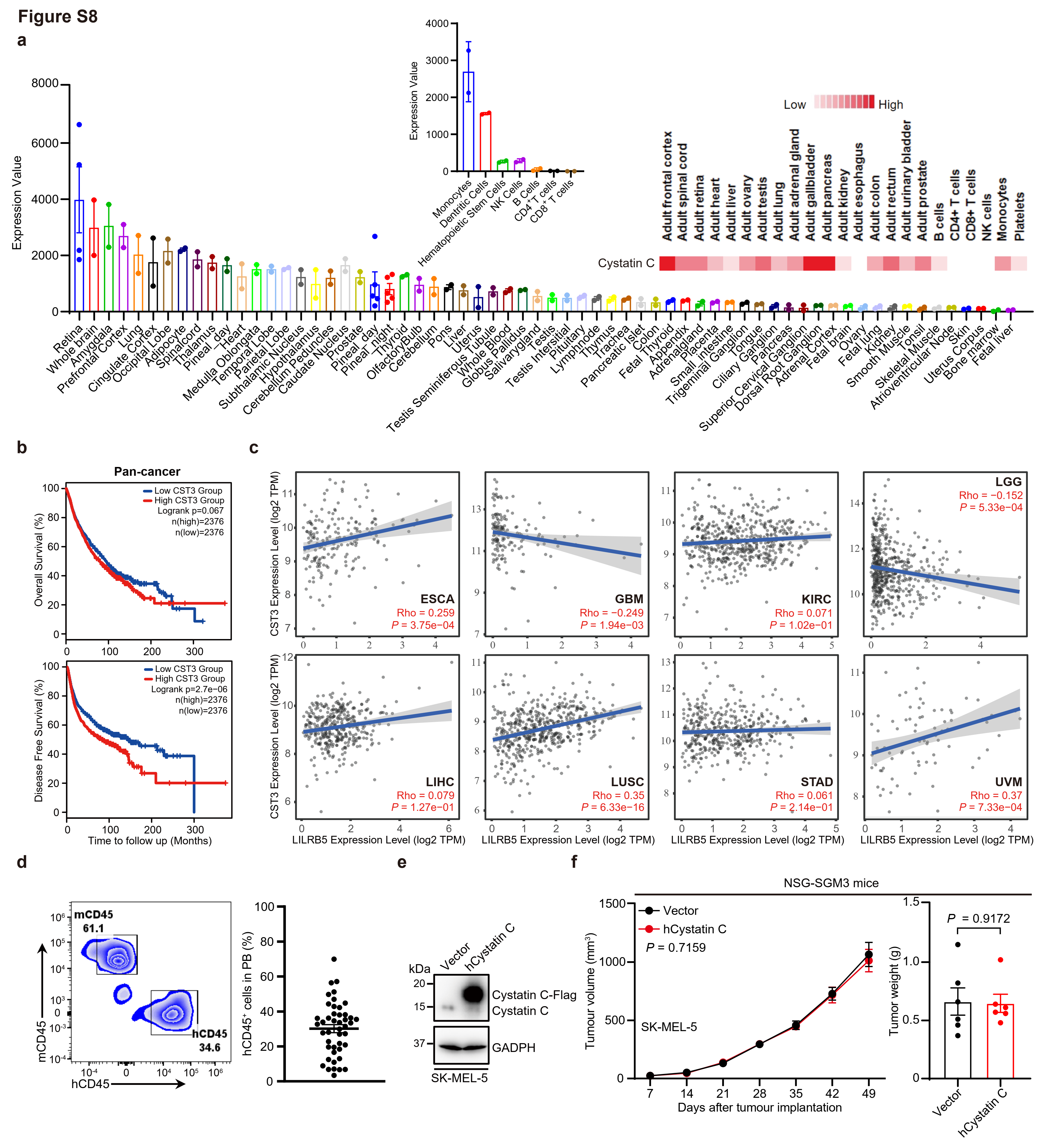


**Fig. S8 Cystatin C in human cancers is associated with a poor outcome, related to Fig. 6.**

1. *CST3* mRNA expression in human tissues and hematopoietic cells (embedded) as indicated in BioGPS microarray database. Cystatin C protein expression in human proteome map mass spectrometry database (embedded).
2. Kaplan–Meier survival analysis of correlations between *CST3* expression and overall survival (Top) or disease-free survival (Bottom) across all cancer patients from the TCGA database. Patients were stratified with low levels of *CST3* (blue) or high levels of *CST3* (red) from the TCGA database. Statistical significance was calculated by log-rank test.
3. Bioinformatic analysis of the correlation between *CST3* and LILRB5 expression across the indicated cancer types was performed using TIMER2.0. For each tumor type, RNA-seq expression values (TPM, Transcripts Per Million) from TCGA were log2-transformed, and Spearman’s rank correlation coefficients (Rho) were calculated. Statistical significance was assessed by *P*-values provided by TIMER2.0, with multiple-testing correction across cancer types using the Benjamini–Hochberg method. Correlations were defined as positive when Rho > 0 with P < 0.05, and negative when Rho < 0 with P < 0.05.
4. Left: Representative flow cytometry pseudo color of hCD45^+^ cell population in peripheral blood of humanized mice. Right: Percentage of hCD45^+^ cell population in peripheral blood of humanized mice (*n* = 45) as measured by flow cytometry.
5. Western blot analysis of human cystatin C in SK-MEL-5 cells transfected with either vector plasmid or *hCST3-Flag* plasmid. GADPH served as the internal control.
6. *SK-MEL-5-vector* or *SK-MEL-5-hcystatin C* melanoma cells were inoculated subcutaneously into NSG-SGM3 mice. Tumor growth curve and tumor weight in each group (*n* = 6) were shown. Data are presented as means ± SEM. *P* values were determined by two-tailed Student’s *t* test.


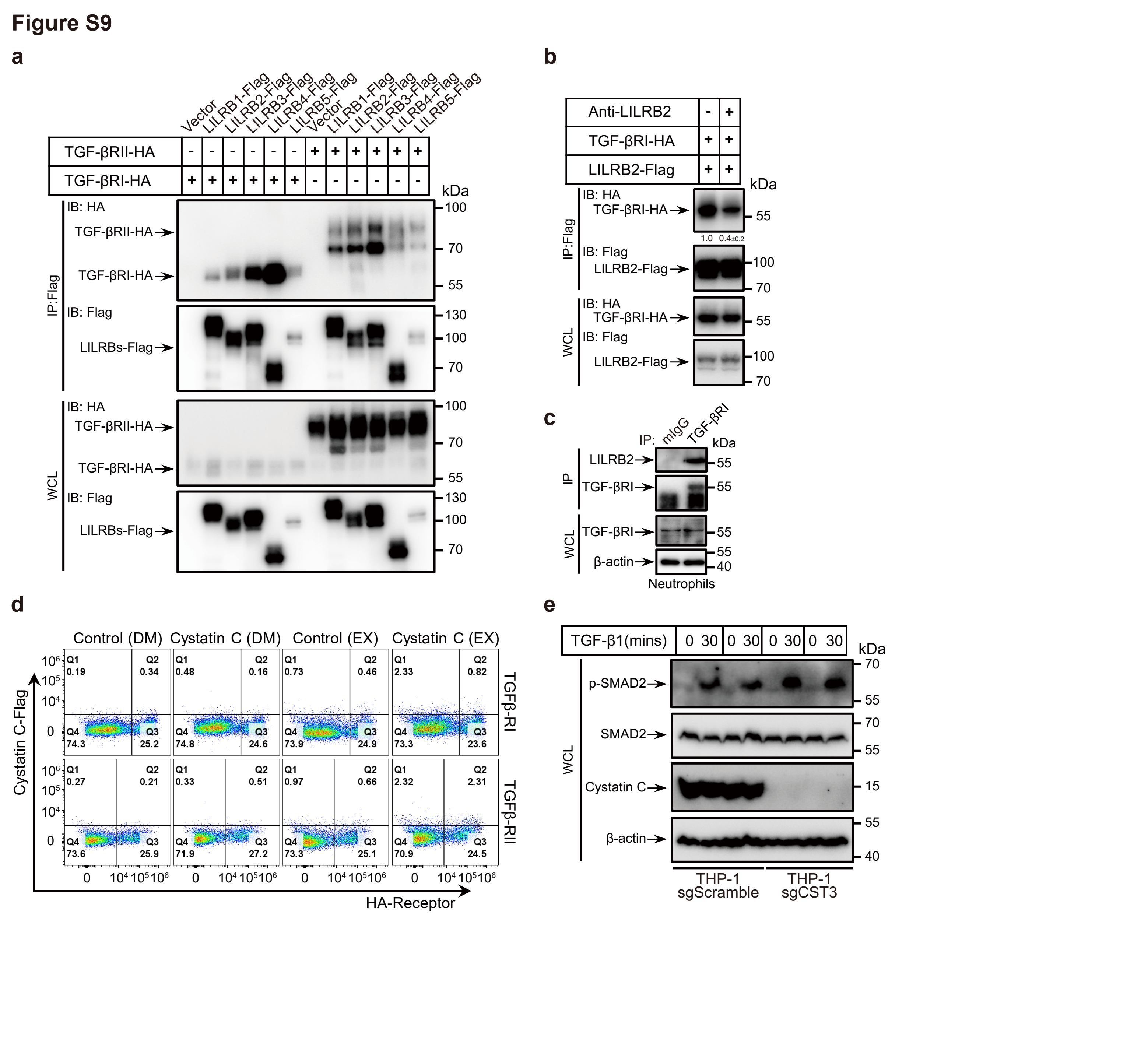


**Fig. S9 Cystatin C inhibits TGF-β signaling in THP-1 cells under physiological conditions, related to Fig. 7.**

1. Co-IP assay showed that LILRBs bind to both TGF-βRI and TGF-βRII in cotransfected HEK293T cells.
2. Co-IP assay showed that Anti-LILRB2 decreases the interactions between LILRB2 and TGF-βRI in cotransfected HEK293T cells. Band intensities were quantified relative to input and are presented as means ± SD. *n* = 3 biological replicates.
3. Co-IP assay showed that LILRB2 binds to TGF-βRI in primary neutrophils. mIgG served as control antibody.
4. Flow cytometry analysis of cystatin C-Flag in DMEM- or EX-CELL-conditioned medium binding to HEK293T cells expressing TGF-βRI or TGF-βRII.
5. Western blot analysis of p-SMAD2, SMAD2, and cystatin C in *THP-1 sgScramble* and *THP-1 sgCST3* cells after overnight starvation and a 30-minutes treatment of TGF-β1 (20 ng/mL). β-actin served as the internal control.


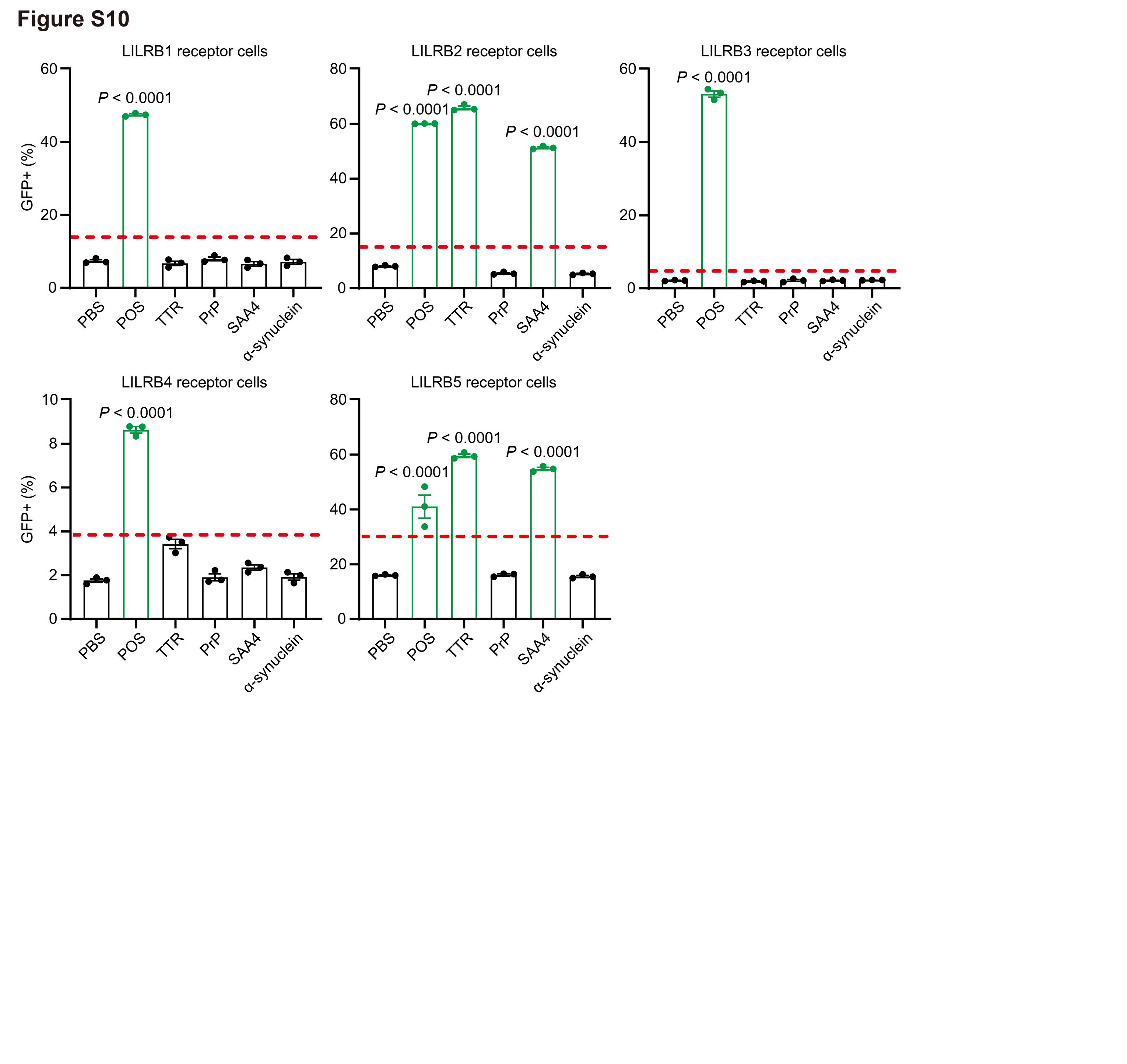


**Fig. S10 TTR and SAA4 activate LILRB2 and LILRB5 reporter cells, related to Fig. 8.**

Percentage of LILRB1, LILRB2, LILRB3, LILRB4, or LILRB5 reporter cells activated on plates coated with TTR (20 μg/mL), PrP (20 μg/mL), SAA4 (20 μg/mL), and α-synuclein (20 μg/mL). *n* = 3 biological replicates. Data are presented as means ± SD. The threshold of activation is defined as twice that of negative control treatment. *P* values were determined by one-way ANOVA with a Dunnett’s multiple comparisons test.


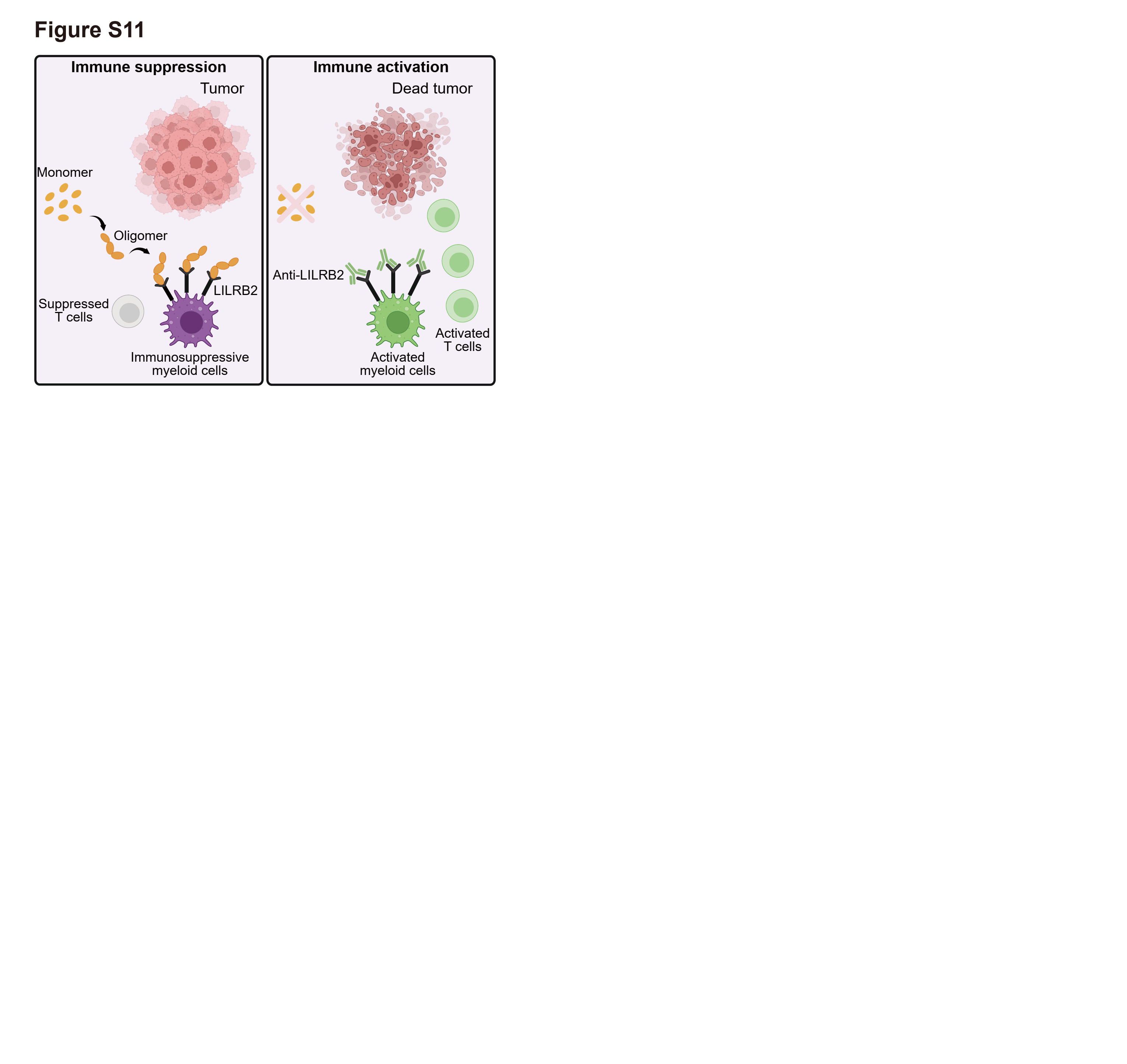


**Fig. S11 Oligomeric cystatin C accelerates tumor development.**

Oligomeric cystatin C supports immunosuppressive activities of myeloid cells through LILRB2 and LILRB5 receptors, resulting in suppression of T cell functions and promotion of cancer progression. Targeting cystatin C oligomerization or blocking LILRB2 and LILRB5 signaling represents a promising strategy for cancer immunotherapy. Figure was generated with BioRender (<https://app.biorender.com/>).

**Supplementary Tables**

**Table S1. Information of blood samples from healthy donors and cancer patients, related to Fig. S1.**

| **Sample Number** | **Sample ID** | **Sample Type** |
| --- | --- | --- |
| 1 | H4522 (Sigma) | Healthy human serum |
| 2 | 35-060-CL (Corning) | Healthy human serum |
| 3 | Carter BloodCare | Healthy human serum |
| 4 | Carter BloodCare | Healthy human serum |
| 5 | Carter BloodCare | Healthy human serum |
| 6 | Carter BloodCare | Healthy human serum |
| 1 | 48728 | Renal mass suspicious for RCC |
| 2 | 48729 | RCC |
| 3 | 48730 | Renal mass suspicious for RCC |
| 4 | 49307 | Lung cancer |
| 5 | 49435 | Renal mass suspicious for RCC |
| 6 | 49438 | Lung adenocarcinoma |
| 7 | 49447 | Renal cancer |
| 8 | 49436 | Renal mass suspicious for RCC |
| 9 | 49578 | Renal mass suspicious for RCC |
| 10 | 49585 | Lung adenocarcinoma |
| 11 | 49586 | Lung cancer |
| 12 | 49702 | Renal cyst suspicious for RCC |
| 13 | 49720 | Lung adenocarcinoma |
| 14 | 49725 | Renal mass suspicious for RCC |
| 15 | 46785 | Lung adenocarcinoma |
| 16 | 48726 | Renal mass suspicious for RCC |
| 17 | 48727 | RCC |
| 18 | 49456 | Renal mass suspicious for RCC |
| 19 | 49599 | Renal mass suspicious for RCC |
| 20 | 49880 | Renal mass suspicious for RCC |
| 21 | 49902 | Lung adenocarcinoma |
| 22 | 49906 | Renal mass suspicious for RCC |
| 23 | 49987 | Lung adenocarcinoma |
| 24 | 49993 | Renal mass suspicious for RCC |
| 25 | 50058 | Renal mass suspicious for RCC |
| 26 | 46784 | Lung adenocarcinoma |
| 27 | 50132 | Melanoma (recurrent) |
| 28 | 50302 | Renal mass suspicious for RCC |
| 29 | 50176 | Metastatic melanoma |
| 30 | 50448 | RCC |
| 31 | 46809 | Renal mass suspicious for RCC |
| 32 | 50509 | ccRCC |
| 33 | 50376 | Renal mass suspicious for RCC |
| 34 | 50379 | SCC of lung |
| 35 | 50381 | Breast cancer (IDC) |
| 36 | 50474 | Renal mass suspicious for RCC |
| 37 | 50598 | Breast cancer (ILC) |
| 38 |  | Leukemia |
| 39 | 50578 | Renal masses |
| 40 | 50633 | Breast cancer/IDC |
| 41 | 50635 | Breast cancer/IDC |
| 42 | 50583 | Lung Squamous cell carcinoma |
| 43 | 50611 | Renal mass suspicious for RCC |

**Table S2. Information of melanoma tumor tissue samples, related to Fig. S1.**

| **Sample Number** | **Sample ID** | **Sample Type** |
| --- | --- | --- |
| 1 | 42273 | Melanoma |
| 2 | 42275 | Melanoma |
| 3 | 43128 | Melanoma |
| 4 | 43695 | Melanoma |
| 5 | 45259 | Melanoma |
| 6 | 45625 | Melanoma |
| 7 | 49031 | Melanoma |
| 8 | 50328 | Melanoma |
| 9 | 20708 | Melanoma |
| 10 | 22010 | Melanoma |
| 11 | 23781 | Melanoma |
| 12 | 26101 | Melanoma |
| 13 | 42272 | Melanoma |
| 14 | 43406 | Melanoma |
| 15 | 43163 | Melanoma |
| 16 | 43467 | Melanoma |

**Table S3. Flow cytometry antibodies.**

| **Marker** | **Fluorescent Tag** | **Species** | **Supplier** | **Catalog#** |
| --- | --- | --- | --- | --- |
| CD45 | Alexa Fluor 532 | Human | Thermo Fisher Scientific | 58-0459-42 |
| CD45 | Pacific Blue | Human | BioLegend | 368540 |
| CD11b | Brilliant Violet 421 | Human | BioLegend | 393114 |
| CD14 | eFluor 506 | Human | Thermo Fisher Scientific | 69-0149-42 |
| CD15 | PerCP-eFluor 710 | Human | Thermo Fisher Scientific | 46-0159-42 |
| CD3 | FITC | Human | BioLegend | 300306 |
| CD4 | Brilliant Violet 570 | Human | BioLegend | 317445 |
| CD8 | APC/Fire 810 | Human | BioLegend | 344764 |
| CD19 | Brilliant Violet 510 | Human | BioLegend | 363020 |
| CD56 | PerCP-eFluor 710 | Human | Thermo Fisher Scientific | 46-0567-42 |
| LILRB1 | PE | Human | Thermo Fisher Scientific | 12-5129-42 |
| LILRB2 | PE | Human | BioLegend | 338706 |
| LILRB3 | PE | Human | BioLegend | 337704 |
| LILRB4 | PE | Human | BioLegend | 333008 |
| LILRB5 | PE | Human | Cheng Cheng Zhang and Zhiqiang An Labs |  |
| CD40 | FITC | Human | BioLegend | 334306 |
| CD80 | APC | Human | BioLegend | 305220 |
| CD86 | Brilliant Violet 711 | Human | BioLegend | 305440 |
| CD16 | PE/Cyanine5 | Human | BioLegend | 302010 |
| CD163 | Brilliant Violet 421 | Human | BioLegend | 333612 |
| CD206 | FITC | Human | BioLegend | 321104 |
| CD62L | FITC | Human | BioLegend | 304804 |
| CD66b | PE | Human | BioLegend | 305117 |
| CD182 | PE | Human | BioLegend | 320706 |
| CD66b | APC | Human | BioLegend | 562586 |
| Phospho-Smad2/Smad3 | PE | Human | BD Biosciences | 562586 |
| PIR-A/B | PE | Mouse | BioLegend | 144104 |
| gp49B | PE | Mouse | BioLegend | 144903 |
| CD45 | Alexa Fluor 532 | Mouse | Thermo Fisher Scientific | 58-0451-82 |
| CD11b | Super Brigh 645 | Mouse | Thermo Fisher Scientific | 64-0112-82 |
| F4/80 | Alexa Fluor 660 | Mouse | Thermo Fisher Scientific | 606-4801-82 |
| CD206 | APC-eFluor 780 | Mouse | Thermo Fisher Scientific | 47-2061-82 |
| Ly6C | APC/Fire 810 | Mouse | BioLegend | 128056 |
| Ly6G | Brilliant Violet 480 | Mouse | Thermo Fisher Scientific | 414-9668-82 |
| CD11c | PE/Cyanine5 | Mouse | BioLegend | 117316 |
| MHC Class II (I-A/I-E) | Super Bright 780 | Mouse | Thermo Fisher Scientific | 78-5321-82 |
| CD80 | APC/Fire 750 | Mouse | BioLegend | 104740 |
| CD14 | Brilliant Violet 421 | Mouse | BioLegend | 123329 |
| STAT3 Phospho (Tyr705) | FITC | Mouse | BioLegend | 651020 |
| CD3 | Alexa Fluor 700 | Mouse | BioLegend | 100216 |
| CD4 | eFluor 506 | Mouse | Thermo Fisher Scientific | 69-0042-82 |
| CD8 | APC-eFluor 780 | Mouse | Thermo Fisher Scientific | 47-0081-82 |
| CD19 | PE | Mouse | BioLegend | 152408 |
| NK1.1 | Super Bright 702 | Mouse | Thermo Fisher Scientific | 67-5941-82 |
| CD25 | Brilliant Violet 421 | Mouse | BioLegend | 102034 |
| IFN-γ | APC | Mouse | BioLegend | 505810 |
| Perforin | FITC | Mouse | BioLegend | 154310 |
| Granzyme B | Pacific Blue | Mouse | BioLegend | 515408 |
| Flag-Tag | PE |  | BioLegend | 637310 |
| Rat IgG2a | PE |  | BioLegend | 400508 |
| HA-Tag | APC |  | BioLegend | 901524 |
| His-Tag | APC |  | R＆D system | IC050A |

**Table S4. Western blot antibodies.**

| **Antibody** | **Species Reactivity** | **Supplier** | **Catalog#** |
| --- | --- | --- | --- |
| Anti-Cystatin C | Human/Mouse | abcam | ab109508 |
| Anti-Human-IgG-Fc |  | Jackson ImmunoResearch | 109-035-008 |
| Anti-phospho-tyrosine (PY20) | Human/Mouse | Santa Cruz Biotechnology | sc-508 |
| Anti-phospho-tyrosine (4G10) | Human/Mouse | Cell Signaling Technology | 96215S |
| Anti-SHP-1 | Human/Mouse | Santa Cruz Biotechnology | sc-7289 |
| Anti-SHP-2 | Human/Mouse | Santa Cruz Biotechnology | sc-7384 |
| Anti-β-actin | Human/Mouse | Cell Signaling Technology | 4970S |
| Anti-GADPH | Human/Mouse | Proteintech | 60004-1-Ig |
| Anti-SMAD2 | Human/Mouse | Cell Signaling Technology | 5339S |
| Anti-phospho-SMAD2 | Human/Mouse | Cell Signaling Technology | 3108S |
| Anti-phospho-p38 | Human/Mouse | Cell Signaling Technology | 9211S |
| Anti-phospho-ERK | Human/Mouse | Cell Signaling Technology | 9101S |
| Anti-phospho-AKT | Human/Mouse | Cell Signaling Technology | 4058S |
| Anti-TGFβ-RI | Human | Santa Cruz Biotechnology | sc-518018 |
| Anti-Transthyretin | Human | abcam | ab92469 |
| Anti-LILRB2 | Human | Cheng Cheng Zhang and Zhiqiang An Labs |  |
| Anti-Flag |  | MBL | M185-7 |
| Anti-HA |  | BioLegend | 901519 |

**Table S5. gRNA sequence for human and mouse *CST3* gene.**

| **Primers** | **Sequence (5' to 3')** |
| --- | --- |
| Scrambled gRNA | CACCGGCCCCGCCGCCCTCCCCTCCGTTT |
| Mouse *CST3* gRNA #1 | CACCTCCAGATCTACAGCGTGCCCGTTT |
| Mouse *CST3* gRNA #2 | CACCGTACCACAGCCGCGCCATACGTTT |
| Human *CST3* gRNA | CACCGAGGCCCCATGGACGCCAGCG |

**Table S6. qPCR primers.**

| **Primers** | **Sequence (5' to 3')** |
| --- | --- |
| Mouse TNF-α RT F | GGTGCCTATGTCTCAGCCTCTT |
| Mouse TNF-α RT R | GCCATAGAACTGATGAGAGGGAG |
| Mouse IL-1β RT F | TGGACCTTCCAGGATGAGGACA |
| Mouse IL-1β RT R | GTTCATCTCGGAGCCTGTAGTG |
| Mouse IL-12A RT F | ACGAGAGTTGCCTGGCTACTAG |
| Mouse IL-12A RT R | CCTCATAGATGCTACCAAGGCAC |
| Mouse IL-12B RT F | TTGAACTGGCGTTGGAAGCACG |
| Mouse IL-12B RT R | CCACCTGTGAGTTCTTCAAAGGC |
| Human IL-10 RT F | TCTCCGAGATGCCTTCAGCAGA |
| Human IL-10 RT R | TCAGACAAGGCTTGGCAACCCA |
| Human NOS2 RT F | GCTCTACACCTCCAATGTGACC |
| Human NOS2 RT R | CTGCCGAGATTTGAGCCTCATG |
