# Supplementary figures and images for: Oligomeric cystatin C supports the immunosuppressive activity of myeloid cells through interaction with inhibitory receptors
Source: Signal Transduct Target Ther. 2025 Nov 14;10:368. doi: 10.1038/s41392-025-02462-x (PMC12615798; doi:10.1038/s41392-025-02462-x)

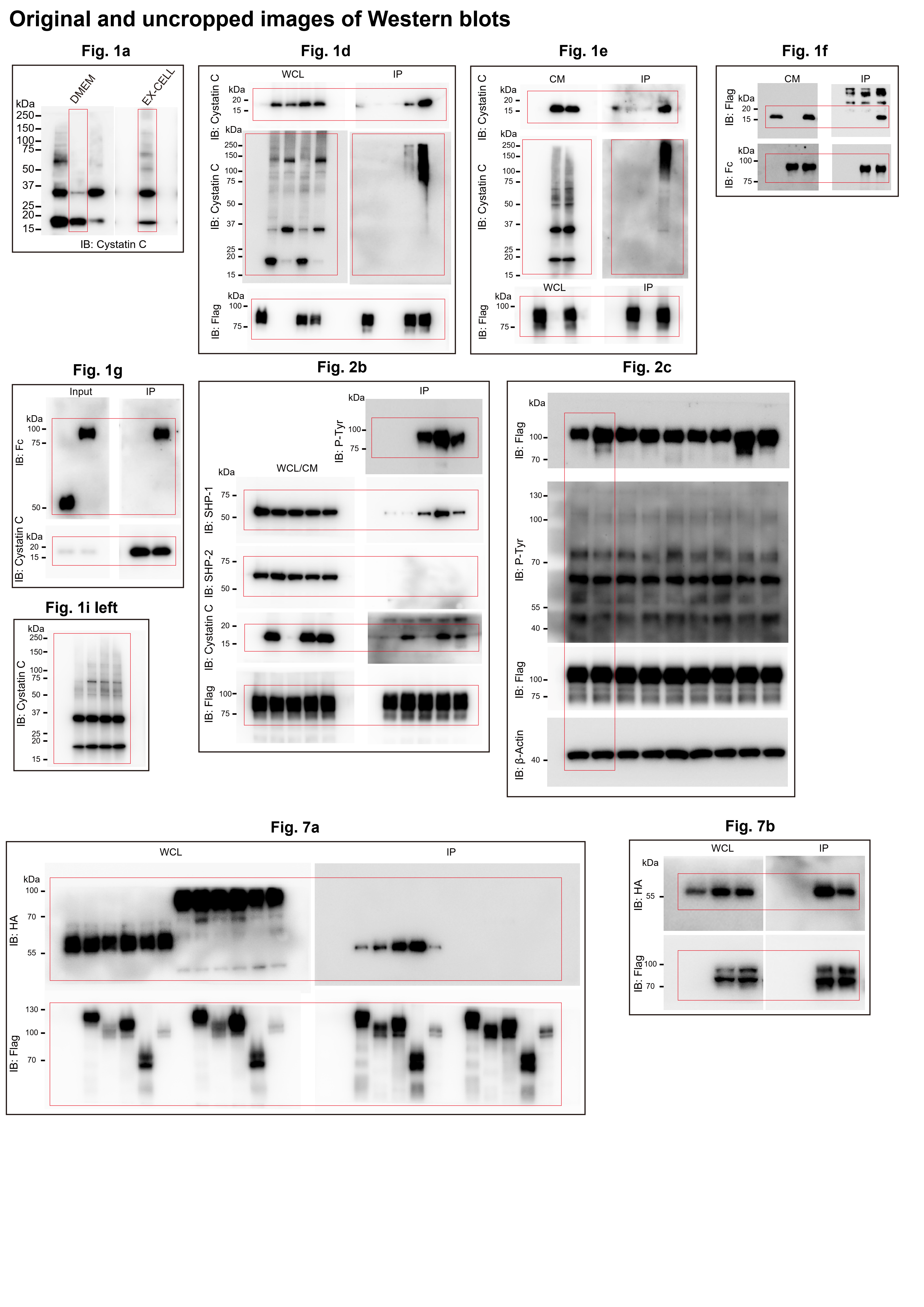

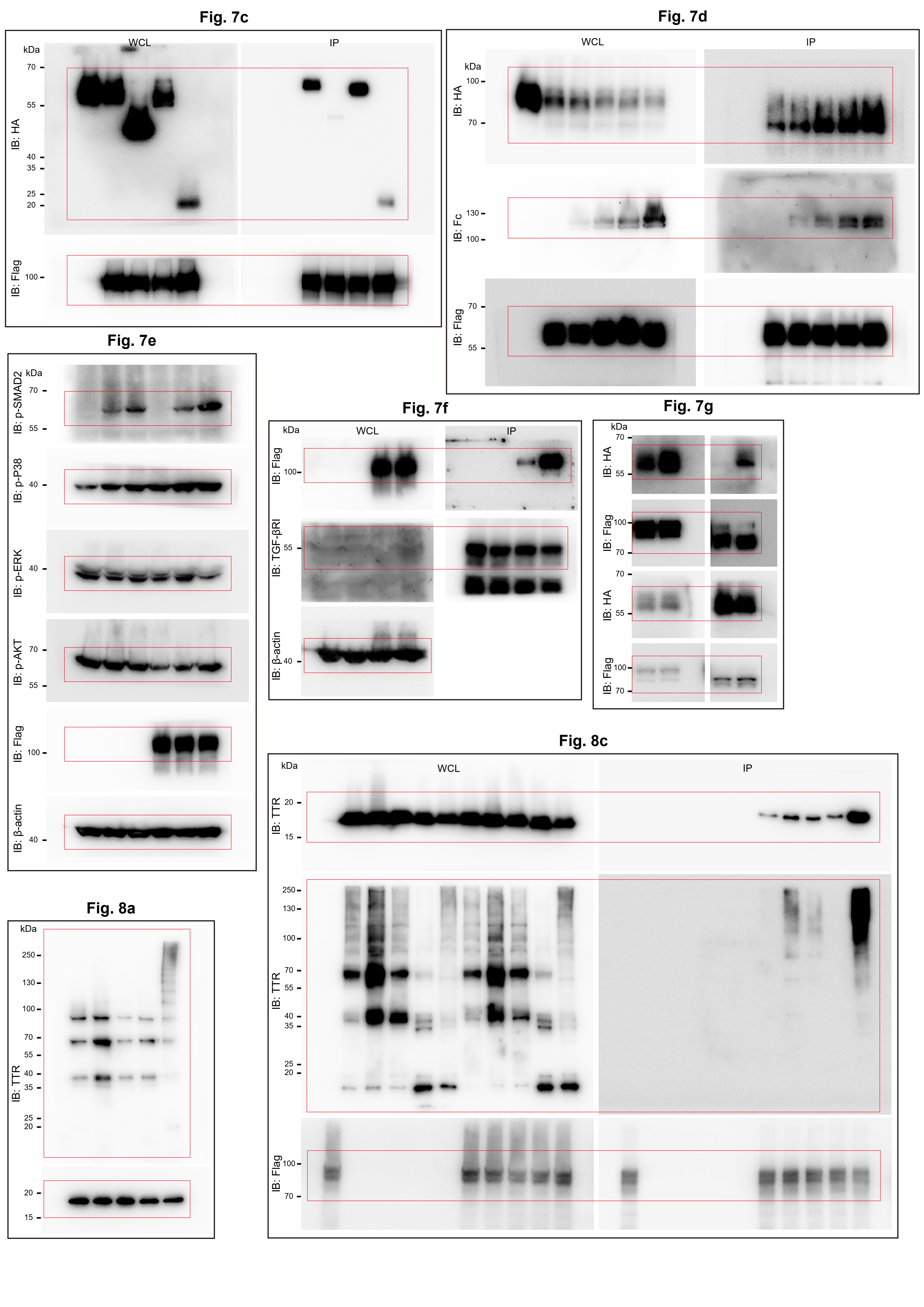

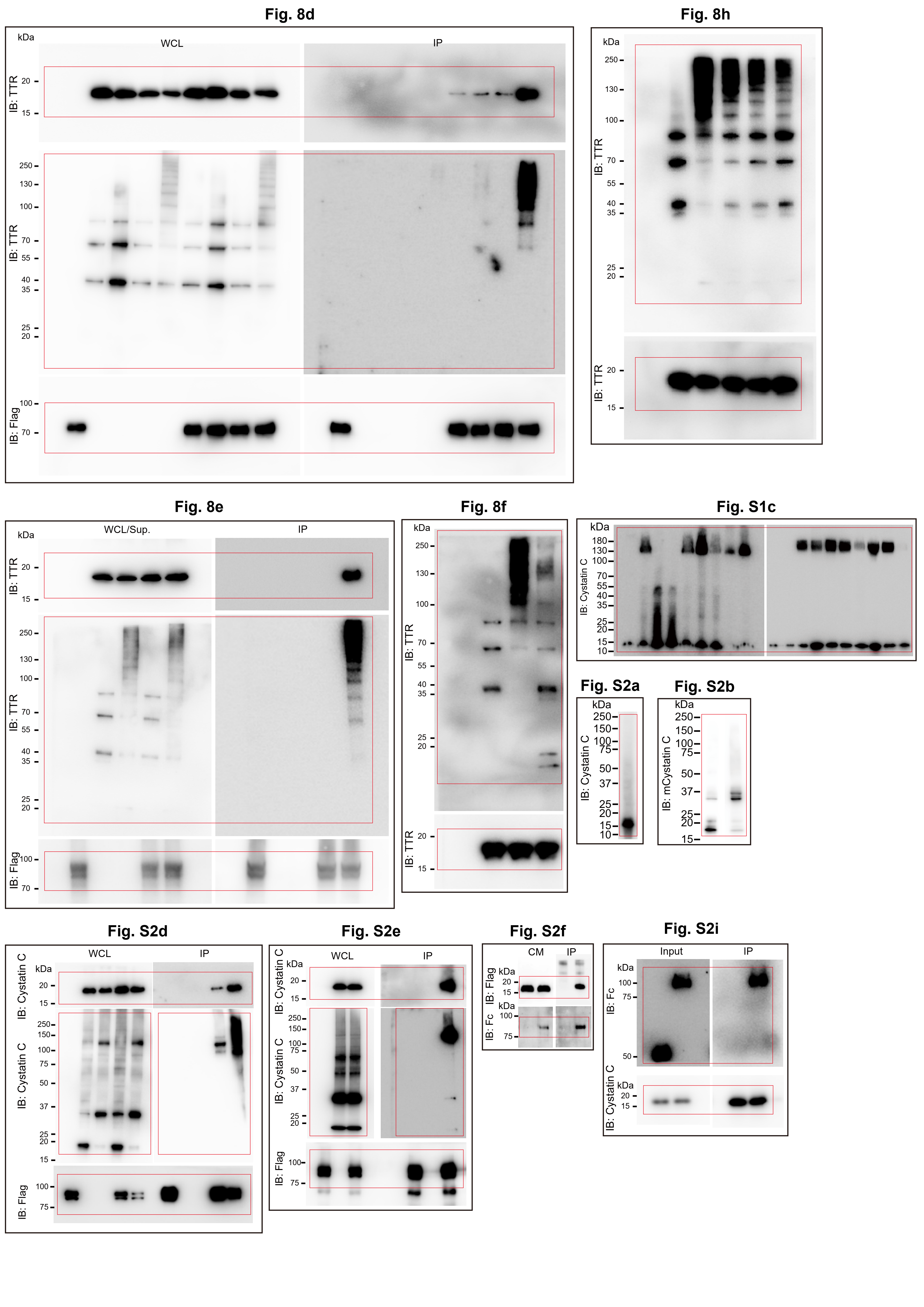

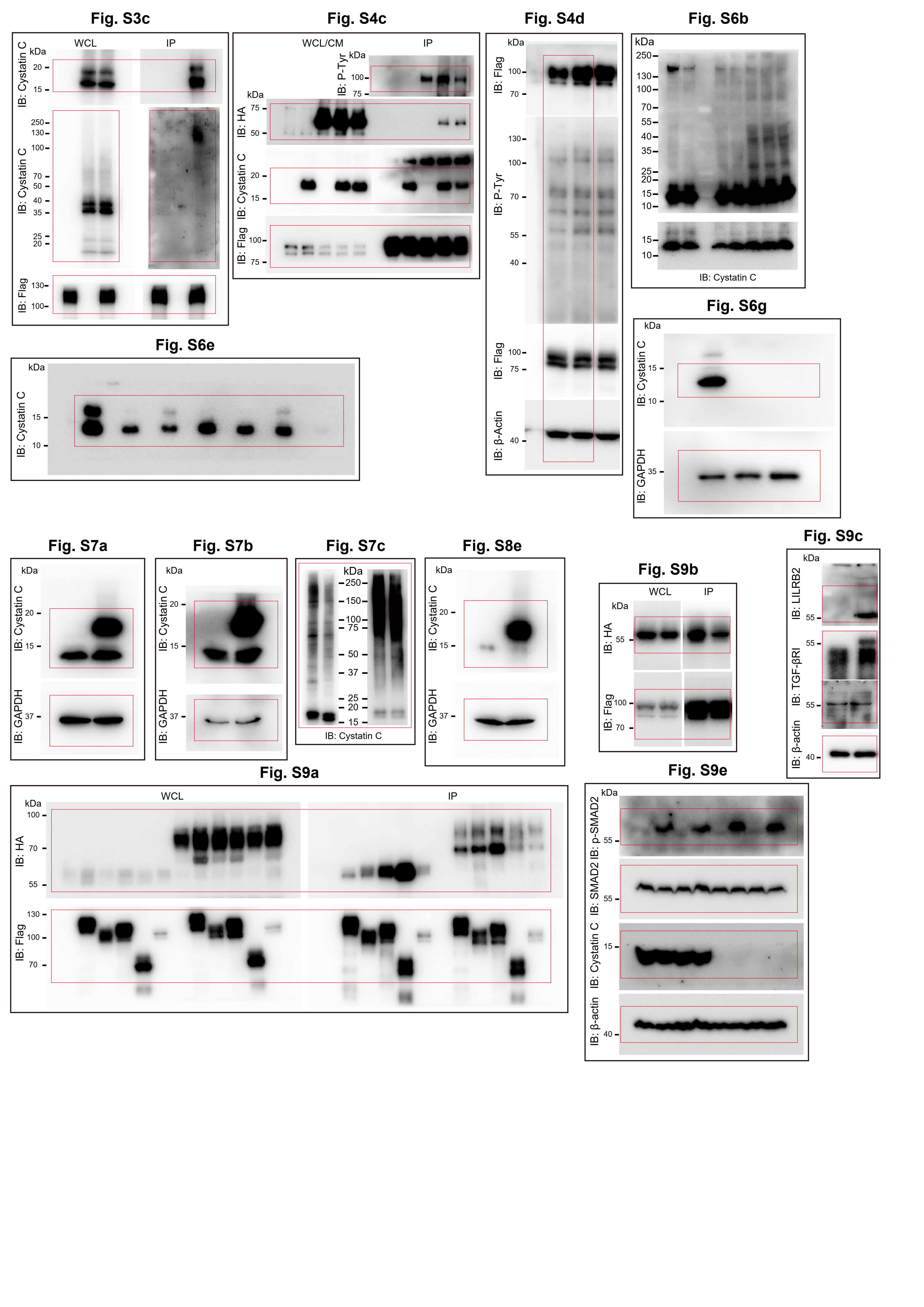

Supplement: Supplementary file 2 — Original and uncropped images of Western blots [file 41392_2025_2462_MOESM2_ESM.docx]
